# Correction: Host-Specific Parvovirus Evolution in Nature Is Recapitulated by *In Vitro* Adaptation to Different Carnivore Species

**DOI:** 10.1371/journal.ppat.1004586

**Published:** 2014-12-05

**Authors:** The PLOS Pathogens Staff

## Notice of Republication

This article was republished on November 20, 2014 to correct typographical errors in Table 1, which were introduced during the typesetting process. Please download this article again to view the correct version. The originally published, uncorrected article and the republished, corrected article are provided here for reference.

## Supporting Information

File S1 Originally published, uncorrected article.
PDF

File S2 Republished, corrected article.
PDF

## References

[1] Allison AB, Kohler DJ, Ortega A, Hoover EA, Grove DM, et al (2014) Host-Specific Parvovirus Evolution in Nature Is Recapitulated by *In Vitro* Adaptation to Different Carnivore Species. PLoS Pathog 10(11): e1004475 doi:10.1371/journal.ppat.1004475 25375184 10.1371/journal.ppat.1004475 PMC4223063

