# Supplementary figures and images for: Host-Specific Parvovirus Evolution in Nature Is Recapitulated by In Vitro Adaptation to Different Carnivore Species
Source: PLoS Pathog. 2014 Nov 6;10(11):e1004475. doi: 10.1371/journal.ppat.1004475 (PMC4223063; doi:10.1371/journal.ppat.1004475)

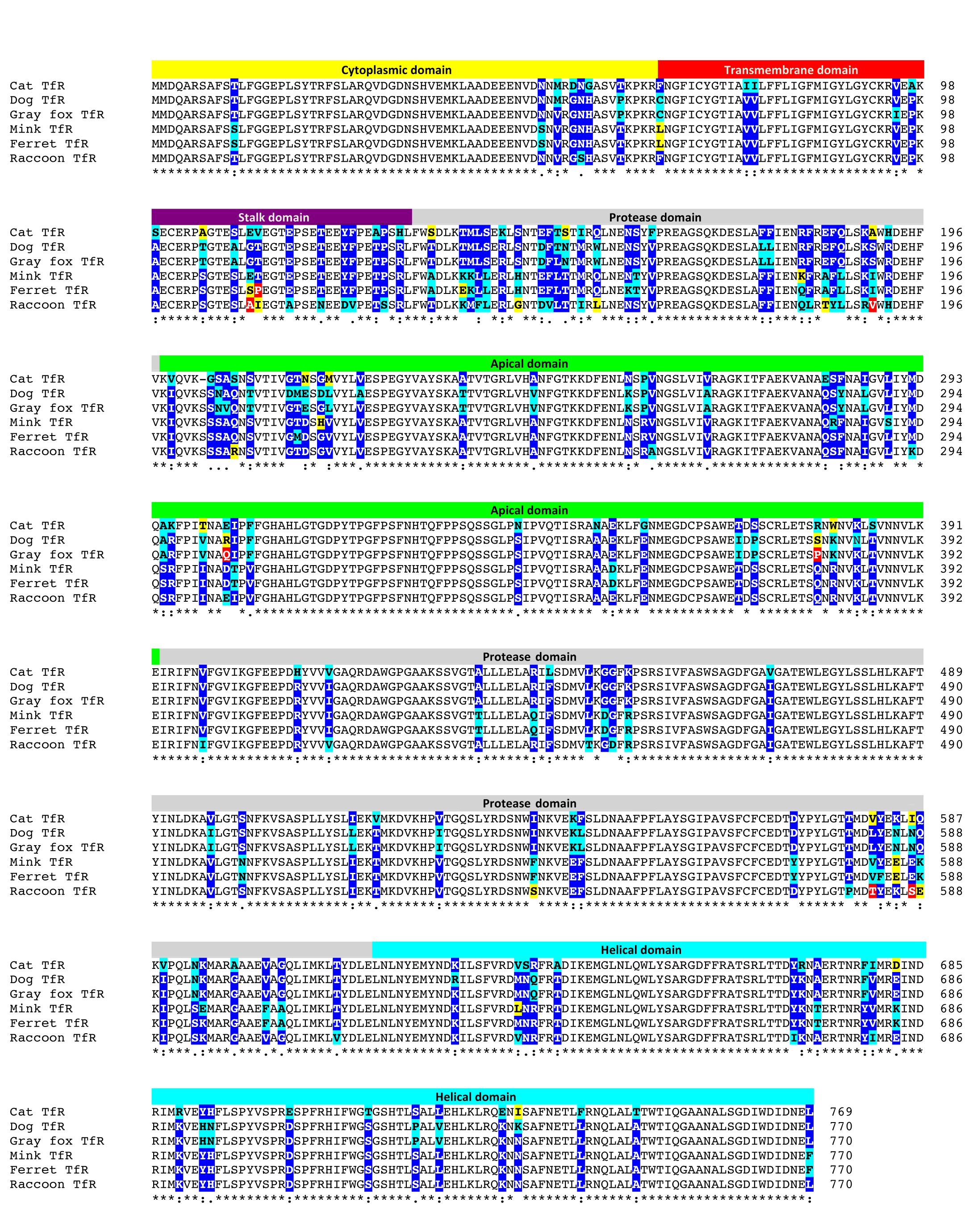

Supplement: Figure S1 — Amino acid alignment of the complete TfRs of the six carnivore hosts used in the experimental evolution studies. The six domains of the TfR (cytoplasmic, transmembrane, stalk, protease, apical, and helical) [17] are indicated atop the alignment. Residues that are variable are highlighted, with each alternate residue shown in royal blue, cyan, yellow, or red. See Table S2 for GenBank accession numbers of each host TfR. (TIF) [file ppat.1004475.s001.tif]

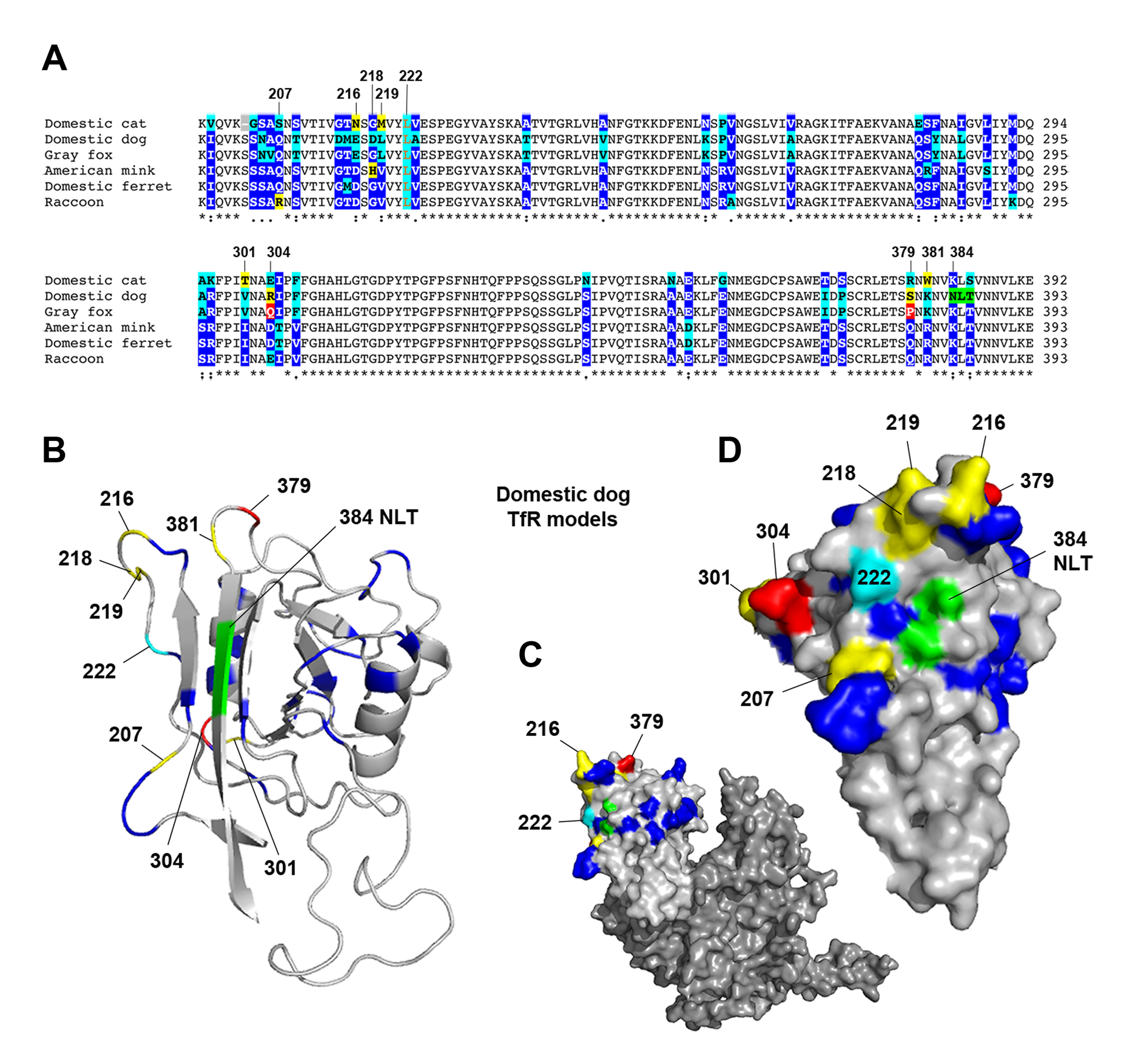

Supplement: Figure S2 — Amino acid alignment of the TfR apical domains of the six carnivore hosts used in the experimental evolution studies and the putative structural location of changes in the domestic dog model of the apical domain. (A) Amino acid alignment focusing on residues 197–393 (based on domestic dog numbering) which constitutes the apical domain, the region of the ectodomain involved in parvovirus binding [18]. Residues that are variable are highlighted, with each alternate residue shown in royal blue, cyan, yellow, or red. Positions that have either three or four different residues among the six species are numbered. The entire column for the 222-Leu residue, shown to be critical in parvovirus binding [21], is highlighted in cyan. The glycosylation site (NLT) at residue 384 in the domestic dog, coyote, and gray wolf TfRs that blocks FPV binding is highlighted in bright green. (B) Structural mapping of the sequence changes in the TfRs of the six hosts analyzed. The ribbon model of the domestic dog apical domain is shown, with residues of divergence among the six hosts color-coded as in panel A, with sites with two, three, or four mutations shown in royal blue, yellow, and red, respectively. (C) Surface rendition of a monomer of the domestic dog TfR homodimer, with the apical domain highlighted in light grey. (D) Side view (∼45° counterclockwise rotation from C) of the apical domain highlighting areas of divergence and/or importance. (TIF) [file ppat.1004475.s002.tif]

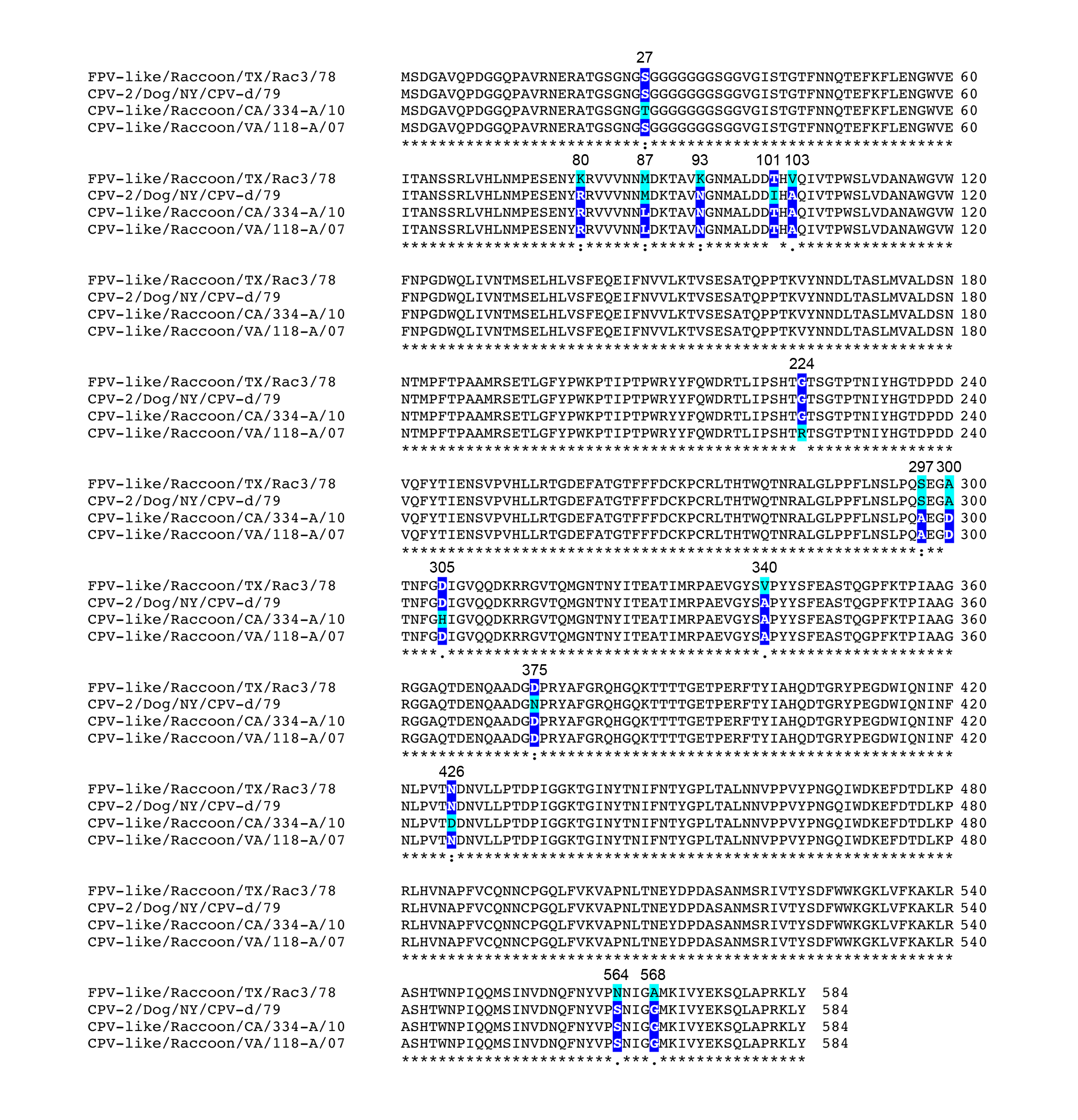

Supplement: Figure S3 — Amino acid alignment of the VP2 protein of the parvoviruses used in the experimental evolution studies (CPV-d, Rac3, Rac118, Rac334). Non-passaged (original) sequences are shown, with residues of divergence highlighted. (TIF) [file ppat.1004475.s003.tif]

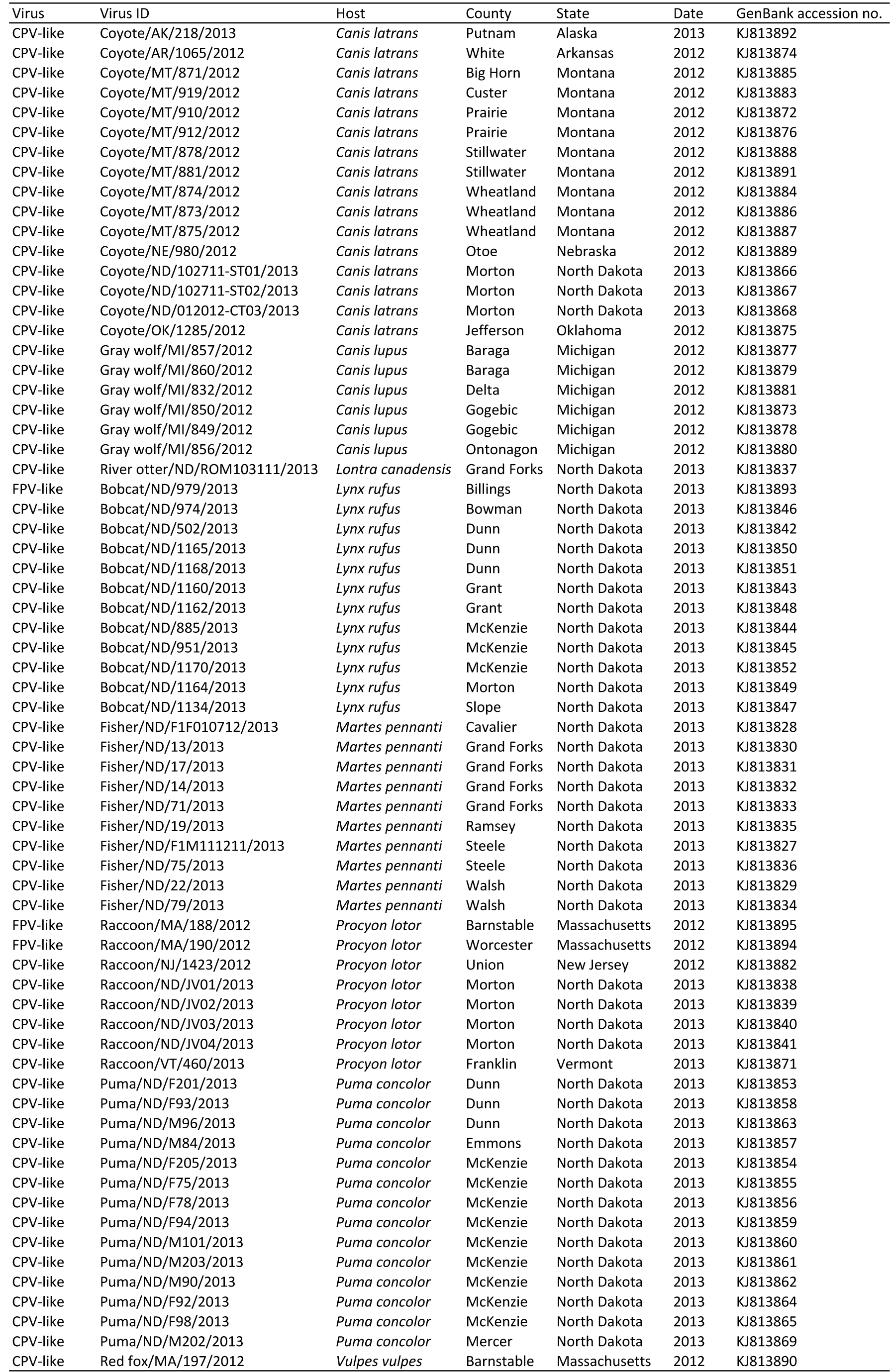

Supplement: Table S1 — New full-length VP2 sequences of parvoviruses obtained from wild carnivores during this study. For each virus, the identification (ID) number, host species, county, state, and date of collection is shown, along with a GenBank accession number. (TIF) [file ppat.1004475.s004.tif]

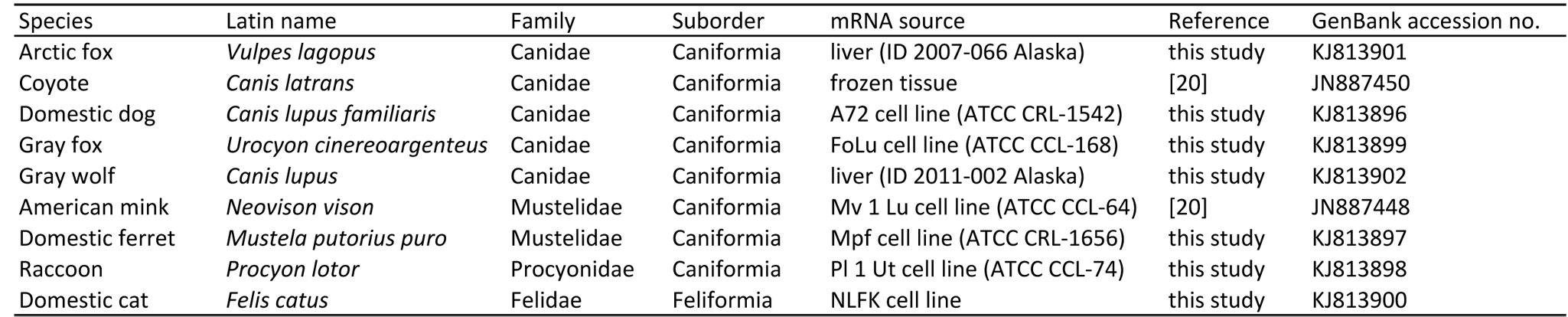

Supplement: Table S2 — New carnivore TfR sequences obtained during this study. For each carnivore species, its taxonomic classification and the source of mRNA is shown, along with a GenBank accession number. (TIF) [file ppat.1004475.s005.tif]
