# Supplementary material for: Correction: Host-Specific Parvovirus Evolution in Nature Is Recapitulated by In Vitro Adaptation to Different Carnivore Species
Source: PLoS Pathog. 2014 Dec 5;10(12):e1004586. doi: 10.1371/journal.ppat.1004586 (PMC4257730; doi:10.1371/journal.ppat.1004586)
Supplement: File S2 — Republished, corrected article. PDF [file ppat.1004586.s002.pdf]

OPEN ACCESS Freely available online
PLOS | PATHOGENS

Host-Specific Parvovirus Evolution in Nature Is Recapitulated by *In Vitro* Adaptation to Different Carnivore Species

Andrew B. Allison[1]*, Dennis J. Kohler[2], Alicia Ortega[1], Elizabeth A. Hoover[1], Daniel M. Grove[3], Edward C. Holmes[4], Colin R. Parrish[1]

1 Baker Institute for Animal Health, Department of Microbiology and Immunology, College of Veterinary Medicine, Cornell University, Ithaca, New York, United States of America, **2** United States Department of Agriculture, Animal and Plant Health Inspection Service, Wildlife Services, National Wildlife Disease Program, Fort Collins, Colorado, United States of America, **3** North Dakota Game and Fish Department, North Dakota State Government, Bismarck, North Dakota, United States of America, **4** Marie Bashir Institute for Infectious Diseases and Biosecurity, Charles Perkins Centre, School of Biological Sciences and Sydney Medical School, University of Sydney, Sydney, New South Wales, Australia

Abstract

Canine parvovirus (CPV) emerged as a new pandemic pathogen of dogs in the 1970s and is closely related to feline panleukopenia virus (FPV), a parvovirus of cats and related carnivores. Although both viruses have wide host ranges, analysis of viral sequences recovered from different wild carnivore species, as shown here, demonstrated that >95% were derived from CPV-like viruses, suggesting that CPV is dominant in sylvatic cycles. Many viral sequences showed host-specific mutations in their capsid proteins, which were often close to sites known to control binding to the transferrin receptor (TfR), the host receptor for these carnivore parvoviruses, and which exhibited frequent parallel evolution. To further examine the process of host adaptation, we passaged parvoviruses with alternative backgrounds in cells from different carnivore hosts. Specific mutations were selected in several viruses and these differed depending on both the background of the virus and the host cells in which they were passaged. Strikingly, these *in vitro* mutations recapitulated many specific changes seen in viruses from natural populations, strongly suggesting they are host adaptive, and which were shown to result in fitness advantages over their parental virus. Comparison of the sequences of the transferrin receptors of the different carnivore species demonstrated that many mutations occurred in and around the apical domain where the virus binds, indicating that viral variants were likely selected through their fit to receptor structures. Some of the viruses accumulated high levels of variation upon passage in alternative hosts, while others could infect multiple different hosts with no or only a few additional mutations. Overall, these studies demonstrate that the evolutionary history of a virus, including how long it has been circulating and in which hosts, as well as its phylogenetic background, has a profound effect on determining viral host range.

Citation: Allison AB, Kohler DJ, Ortega A, Hoover EA, Grove DM, et al. (2014) Host-Specific Parvovirus Evolution in Nature Is Recapitulated by *In Vitro* Adaptation to Different Carnivore Species. PLoS Pathog 10(11): e1004475. doi:10.1371/journal.ppat.1004475

Editor: Raul Andino, University of California San Francisco, United States of America

Received May 15, 2014; **Accepted** September 16, 2014; **Published** November 6, 2014

This is an open-access article, free of all copyright, and may be freely reproduced, distributed, transmitted, modified, built upon, or otherwise used by anyone for any lawful purpose. The work is made available under the Creative Commons CC0 public domain dedication.

Data Availability: The authors confirm that all data underlying the findings are fully available without restriction. All relevant data are within the paper and its Supporting Information files except for the new parvovirus and transferrin receptor sequences, which are available from GenBank under the accession numbers KJ813827-KJ813895 and KJ813896-KJ813902, respectively.

Funding: Research was funded by National Institutes of Health (NIH) grant RO1GM8496821 to CRP and ECH, by NIH grant R01AI092571 to CRP, and by a National Research Service Award Fellowship (F32AI100545) to ABA from the National Institute of Allergy and Infectious Diseases, NIH (http://www.nih.gov/). ECH is additionally supported by a National Health and Medical Research Council Australia Fellowship (https://www.nhmrc.gov.au/). Funding for sampling of wild carnivores was provided by the United States Department of Agriculture-Animal Plant Health Inspection Service-Wildlife Services, National Wildlife Disease Surveillance and Emergency Response Program (http://www.aphis.usda.gov/wps/portal/aphis/ourfocus/wildlifedamage) and by the North Dakota Game and Fish Department (NDGFD) (http://gf.nd.gov/). All NDGFD carnivore tissue samples were collected under studies funded in part and approved by the United States Fish and Wildlife Service Grant Agreements for 2010–2014 Project W-67-R-50, W-67-R-51, W-67-R-52, W-67-R-53; Studies E-II, E-III, E-XI. The funders had no role in study design, data collection and analysis, decision to publish, or preparation of the manuscript

Competing Interests: The authors have declared that no competing interests exist.

* Email: aba75@cornell.edu

Introduction

Host range is a key property of a virus that reflects the diversity of species that it can naturally infect, and expansions in viral host ranges provide the potential for the emergence of new diseases [1]. However, host range is often difficult to define and many factors need to be incorporated, such as the host susceptibility to infection, as well as the ability of the virus to undergo sustained transmission in the new host [2,3]. Here we examine host range variation and its determinants in a number of parvoviruses (genus *Protoparvovirus*, family *Parvoviridae*) that are over 98% identical in nucleotide sequence, and which all derive from common ancestors in the recent past [4]. These viruses naturally infect a variety of hosts within the mammalian order Carnivora and are of particular interest as they exhibit varying host ranges. Parvoviruses of domestic cats (*Felis catus*) and related carnivores [including American mink (*Neovison vison*) and raccoon (*Procyon lotor*)] have been known for many years and are generally described as

Author Summary

Canine parvovirus (CPV) is an important example of a viral pathogen that evolved by cross-species transmission and mutation to initiate a disease pandemic. Carnivore parvoviruses infect many species, and their passage in different hosts may select mutations that facilitate host jumping; for example, natural passage of CPV in raccoons may have facilitated its adaptation to dogs. Conversely, some raccoon-adapted viruses are non-infectious to dogs, illustrating that host range barriers exist among different carnivores. Here we demonstrate that these barriers can be overcome by only a few mutations in the virus that likely alter host receptor binding, and that host adaptation can differ dramatically among very similar viruses. Importantly, we also show that passage of viruses in cell cultures of different hosts results in mutations at the same sites that vary in nature and confer fitness increases, strongly suggesting that they are adaptively important. These findings demonstrate that parvoviruses may cross species barriers to infect less susceptible hosts through single or only a few mutations, and that differences in the genetic background, host range, and/or evolutionary history of the viruses influence their propensity to jump hosts. Overall, these discoveries help reveal the mechanisms that control host switching and viral emergence.

feline panleukopenia virus (FPV) or related variants, while canine parvovirus (CPV) emerged in the 1970s as a new virus (termed CPV-2) that (unlike FPV) could infect domestic dogs (*Canis lupus familiaris*), wolves (*Canis lupus*) and coyotes (*Canis latrans*), and which caused a disease pandemic in 1978 [5]. Around 1980, a new genetic and antigenic variant of CPV emerged (termed CPV-2a), which also spread pandemically and supplanted CPV-2 in dogs worldwide within a few years [6]. In the decades since its emergence, CPV-2a has undergone additional evolution and a number of antigenic and genetic variants are now circulating worldwide [2].

The CPV virion contains 60 copies of VP1 and VP2, and as the entire VP2 sequence is contained within VP1, these proteins appear to constitute equivalent structural units during capsid assembly, although VP2 lacks the N-terminal 143 amino acids of VP1 [7,8]. VP1 and VP2 contain a core structure composed of an eight-stranded, anti-parallel β-barrel motif, where the β-strands are connected by large loops that make up most of the surface of the virus [8]. Around the three-fold axes of symmetry, protruding loop structures are prominent in both eliciting neutralizing antibodies and determining host range [9,10]. CPV gained the ability to infect dogs through mutations in the capsid protein that fell into three regions (defined by VP2 residues 93, 300, and 323) that are exposed on the surface of the virus [9,11–13]. These capsid variations control the ability of the virus to interact with its cellular receptor, the transferrin receptor type-1 (TfR), and binding to the domestic dog TfR was important for the canine host shift [14,15]. The TfR is a homodimeric type II transmembrane protein that transports iron into the cell by binding and internalizing iron-loaded transferrin [16]. Each monomer of the TfR contains a large ectodomain composed of three distinct regions: a protease, helical, and apical domain [17]. Domestic dog and cat TfRs differ by ~12% in amino acid identity, but the presence of a unique glycosylation site in the apical domain of the dog TfR was a primary determinant blocking binding and infection of FPV-like viruses in dogs and related canids [18–20]. However, other changes of residues in the apical domain of the TfR also influence virus binding [21], suggesting that the capsid-receptor interactions are complex and likely vary depending on both the specific host and viral structures.

Historically, most FPV and CPV isolates have been recovered from domestic cats and dogs, although they have periodically been isolated from other carnivores [22–24]. These alternative hosts may exist as free-ranging wild populations, or be domesticated or farmed for fur and/or meat production in much higher numbers and densities than are seen in the wild [e.g., American mink, Arctic ("blue") fox (*Vulpes lagopus*), raccoon dog (*Nyctereutes procyonoides*)] [25–27]. The parvoviruses that exist in the wildlife reservoir may have introduced alternative ecological and evolutionary pathways that facilitated the pandemic emergence of both CPV-2 and CPV-2a in dogs [28]. Indeed, viruses clearly 'intermediate' between FPV and CPV-2 and between CPV-2 and CPV-2a have not been detected in domestic dog or cat populations, suggesting that other hosts may have been involved in the evolution and emergence of these pandemic viruses [29]. Additionally, it is possible that FPV and CPV-2 may have been separately derived from common sylvatic ancestors [28].

The breadth of viral host ranges may be tentatively assessed by the frequency of recovery of field isolates from different hosts, as well as by comparing the phylogenetic relationships between the viruses, which provides insights into whether a virus from a particular host represents a transient spillover or sustained inter-host transmission [28]. Additionally, host potential can be examined through experimental infections to determine susceptibility and transmissibility in various animals. However, when host range barriers are recognized that block infection by a particular virus, the way(s) in which viruses may circumvent these barriers to gain the ability to replicate and spread in a novel host are often obscure.

Here we examine a number of representative parvoviruses and define the mutations in the viral capsid that control infection of different carnivore species. To determine how viruses adapt to alternative hosts, we passaged viruses with varying genetic backgrounds in cells derived from different carnivore species and compared the results to the sequences of viruses recovered from nature. Our rationale is that mutations that are consistently associated with specific hosts in both natural systems and after experimental cell passage are likely to be host range determinants.

Results

Analysis of parvoviruses in nature and host-specific variation

To better define the natural host range of viruses related to CPV and FPV, we tested over 850 individual carnivores encompassing 18 different species in the United States for parvoviral DNA, sequenced the full-length VP2 capsid protein gene (1755nt) from select positive samples, and compared them to other carnivore parvovirus sequences. Our goals were to determine (i) the extent and distribution of CPV-like and FPV-like viruses in wild carnivores, (ii) the roles of different carnivore hosts in parvovirus transmission in the wild, particularly which species are involved in long-term transmission rather than transient spill-over infections, and (iii) whether some mutations were consistently associated with particular carnivore species, suggesting that they influence host specificity.

The carnivore species tested and their state of origin, number of animals positive for CPV or FPV infection, and prevalence rates for each species are shown in Table 1 (a list of the new complete VP2 sequences recovered during the study is shown in Table S1). Animals were randomly sampled in conjunction with ongoing state and federal surveillance and/or nuisance/damage control

Table 1. Parvoviruses recovered from wild carnivores in the United States during this study, indicating the species and number of individual animals tested, the different states sampled, and the prevalence rates of parvovirus DNA detection from tissues.

Species	Latin Name	Family	Suborder	No. positive	No. tested	Prevalence (%)	States tested (no. positive/no. tested)
Gray fox	*Urocyon cinereoargenteus*	Canidae	Caniformia	0	21	0	AL (0/5), MA (0/1), ME (0/3), MN (0/1), NH (0/2), NY (0/2), PA (0/6), VT (0/1)
Arctic fox	*Vulpes lagopus*	Canidae	Caniformia	0	2	0	AK (0/2)
Swift fox	*Vulpes velox*	Canidae	Caniformia	0	1	0	ND (0/1)
Red fox	*Vulpes vulpes*	Canidae	Caniformia	4	98	4.1	AK (0/32), CO (0/5), ID (0/2), MA (2/8), ME (1/2), MN (0/1), MT (0/1), NJ (0/20), NY (0/5), OH (0/3), PA (1/14), UT (0/2), VT (0/3)
Coyote	*Canis latrans*	Canidae	Caniformia	91	382	23.8	AK (1/4), AL (1/10), AR (2/12), CO (7/23), FL (11/26), IL (1/12), IN (0/2), KS (1/11), LA (0/2), MA (0/4), ME (1/11), MI (5/28), MN (3/7), MO (1/5), MT (30/65), NE (7/14), NH (0/4), NJ (0/2), NY (0/11), OH (2/20), OK (2/34), PA (0/21), RI (1/3), TX (2/18), UT (5/12), WY (8/21)
Gray wolf	*Canis lupus*	Canidae	Caniformia	9	21	42.9	MI (9/21)
Striped skunk	*Mephitis mephitis*	Mephitidae	Caniformia	0	9	0	MA (0/4), NJ (0/2), PA (0/2), UT (0/1)
Fisher	*Martes pennanti*	Mustelidae	Caniformia	10	50	20.0	ND (10/50)
American mink	*Neovision vison*	Mustelidae	Caniformia	0	6	0	ID (0/1), OH (0/3), MA (0/2)
North American river otter	*Lontra canadensis*	Mustelidae	Caniformia	1	24	4.2	AL (0/4), IN (0/1), ND (1/19)
American marten	*Martes americana*	Mustelidae	Caniformia	0	3	0	ND (0/3)
Raccoon	*Procyon lotor*	Procyonidae	Caniformia	17	91	18.7	AL (1/3), AR (0/3), IL (0/1), IN (1/4), LA (0/2), MA (2/7), ME (0/7), MO (1/5), ND (4/7), NE (2/9), NH (1/4), NJ (2/11), OH (1/14), PA (0/2), RI (0/2), UT (1/5), VT (1/5)
Alaskan brown bear	*Ursus arctos alascensis*	Ursidae	Caniformia	0	2	0	AK (0/2)
American black bear	*Ursus americanus*	Ursidae	Caniformia	0	1	0	NJ (0/1)
Bobcat	*Lynx rufus*	Felidae	Feliformia	13	82	15.9	AL (0/3), AK (0/1), AR (0/1), ND (13/76), NY (0/1)
Canada lynx	*Lynx canadensis*	Felidae	Feliformia	0	6	0	AK (0/6)
Puma	*Puma concolor*	Felidae	Feliformia	16	24	66.7	ND (16/24)
Small Indian mongoose[a]	*Herpestes auropunctatus*	Herpestidae	Feliformia	0	29	0	HI (0/27), Puerto Rico (0/2)
				161	852	18.9%	

[a]Non-native species; introduced into Hawaii and Puerto Rico [see reference 30].
doi:10.1371/journal.ppat.1004475.t001

programs and none of the animals tested were observed to be actively demonstrating clinical symptoms of parvovirus infection (e.g., diarrhea, hemorrhagic enteritis), suggesting detection of parvoviral DNA likely represented a previous (recovered) infection. Indeed, attempts at isolation of select viruses from DNA-positive tissues did not result in the recovery of live virus.

The overall prevalence of CPV and FPV infection from wild carnivores collected from 30 states, including Alaska and Hawaii, was 18.9% (161/852). In cases where a large number of individual animals of a given species were tested (>20–400), some species (coyote, gray wolf, puma, raccoon, fisher, bobcat) had high infection rates (15.9–66.7%), while the rates of infection for other species (river otter, red fox, gray fox, small Indian mongoose) were much lower (0.0–4.2%) (Table 1) [30]. However, it should be noted that the number of samples from individual species and locations varied. Additionally, we noted some regional variation in infection rates. For example, prevalence rates for parvovirus DNA recovery from coyotes ranged from 0.0% (0/21) in Pennsylvania to 46.2% (30/65) in Montana (Table 1). Despite these possible inherent sampling variabilities, our data clearly demonstrates that many different wild carnivore species in the United States are infected with parvoviruses, and that some hosts are infected at a sufficiently high prevalence to suggest they are experiencing sustained onward transmission in the wild (Figure 1; Table 1). As previously noted [28], we detected a higher prevalence and greater genetic diversity of parvoviruses in large carnivore species such as pumas (Table 1), suggesting that in addition to the well recognized fecal-oral route of infection observed among domestic animals, carnivory (and/or scavenging) may be playing an important role in parvovirus transmission in the wild (Figure 1 inset). Additionally, it was striking that CPV was much more prevalent in wild carnivore populations than FPV, with CPV-like viruses accounting for 98.1% (158/161) of the parvoviruses detected. Even when coyote and gray wolf samples were omitted (i.e., species that are refractory to FPV infection), 95.1% (58/61) of the viruses were of CPV origin, further demonstrating a strong prevalence of CPV infection over FPV in these North American wild carnivore hosts.

A maximum likelihood phylogenetic analysis of 343 full-length VP2 nucleotide sequences further supported the notion of sustained onward transmission in wild carnivores, as well as frequent cross-species transmission (Figure 1; color-coded by host species). In particular, monophyletic groupings of viruses sampled from single wild carnivore species such as coyote, puma, fisher, and raccoon were apparent, strongly suggesting that sustained viral transmission is occurring in these hosts (shown as collapsed clades in Figure 1). In addition, it was striking how frequently these wildlife samples were interleaved with CPV sequences from domestic dogs and cats, suggesting frequent viral traffic between them, albeit with an uncertain directionality in most cases (note the mixing of colors across the tree). Finally, this phylogenetic analysis revealed a remarkable level of parallel evolution at some amino acid sites. This was most apparent at VP2 residue 300 where the Gly-to-Asp mutation has unambiguously evolved five times independently, and at VP2 residue 426, where the Asn-to-Asp mutation has evolved four times, as has the reverse mutation, Asp-to-Asn. Parallel mutation was also observed at a variety of other sites in VP2 with, for example, two independent occurrences of Tyr-to-His at residue 305 and, overall, 33% of all amino acid changes were homoplasious (i.e., shared by sequences but not because of common ancestry).

As the gray wolf, coyote, and domestic dog were identical in their TfR sequences (see below), we next analyzed the amount of VP2 variation that existed in wild coyote and gray wolf populations. If host TfR sequence and structure was the primary determinant in VP2 variation, then most viruses detected in coyotes and wolves should look very similar to domestic dog viruses (unless they were derived through carnivory of other infected hosts) and possibly show mutations at identical residues. Analysis of 100 parvoviruses detected from coyotes or wolves from 26 states demonstrated that they (and no other species) exhibited mutations observed in domestic dogs such as VP2 440-Ala (in association with VP2 426-Glu) [31], in addition to the dog-specific residues VP2 300-Gly and 305-Tyr. Again, our analysis suggests that these mutations have been acquired multiple times in parallel, and hence are likely to be of selective importance. For example, the Thr-to-Ala mutation at residue 440 has occurred six times independently, while the Asp-to-Glu change at residue 426 has evolved three times. By comparing TfR sequence similarity among various carnivores and the viruses recovered from such hosts, it may be possible to determine how the receptor dictates VP2 genetic variation.

Evolutionary analysis of raccoon viruses and their relationship to dog viruses

We have previously shown that CPV-like viruses from raccoons have unique amino acids residues at two key positions – VP2 300 and 305 – that distinguish them from dog viruses (Table 2) [29]. In the current study, we investigated whether raccoon-like and dog-like viruses are being transferred back-and-forth in nature and whether viruses showing signature residues of both dog and raccoon viruses, and possibly in transition between hosts, could be detected. To this end, we recovered a number of CPV-like viruses from raccoons that fell in different phylogenetic positions. Some raccoon viruses clustered phylogenetically with gray wolf and dog viruses and showed VP2 residues characteristic of these viruses (300-Gly and 305-Tyr) without any raccoon-like residues (RacJV01/ND; Figure 1 and Table 2). We also identified viruses in raccoons that possessed the characteristic residues observed in raccoon viruses (e.g., 300-Asp and 305-His), but which still clustered with dog and coyote viruses in the phylogeny (Rac218A/VA; Figure 1 and Table 2), suggesting these were viruses of canid origin that had already experienced evolution at these two key sites. Furthermore, we found viruses that appeared intermediate in this transition, as they possessed both raccoon virus-like (300-Asp) and dog virus-like (305-Tyr) residues (Rac460/VT/12; Figure 1 and Table 2), suggesting recent transfer from dogs (or dog-like canids) to raccoons, consistent with the results of the experimental evolution studies in cell culture (see below).

In vitro passaging and adaptation of parvoviruses to different host cells

The viruses chosen for experimental evolution analysis included (i) an FPV-like virus from raccoons (Rac3), (ii) the prototype pandemic CPV-2 strain (CPV-d) from dogs, and (iii) the prototype CPV-2a-like raccoon virus (Rac118). These viruses were passaged up to 20 weeks in cells from six different carnivore hosts (domestic dog, domestic cat, domestic ferret, American mink, gray fox, and raccoon). Although the six host cell lines were derived from alternate tissue types (e.g., lung, kidney, uterus) which may vary in their TfR expression levels and/or the amounts of virus they produce, all were susceptible to infection (consistent with the pantropic nature of these viruses) and thus provide the most appropriate *in vitro* models for these adaptation studies until a tissue type-specific cell line is developed for multiple different carnivore species. The overall results of the cell culture studies are shown in Figure 2A. We then compared our new and previously sequenced viruses from domestic and wild carnivores in nature

with those mutations observed in the experimental evolution studies to determine which mutations had occurred in parallel (Figure 2B–C). Additional information on the background context of mutations observed in nature versus under experimental conditions is shown in Table 2.

Passage of FPV-like raccoon isolate Rac3 in the six hosts resulted in only a single mutation (VP2 position 556) in one cell line (gray fox) and only after 20 passages (Figure 2A). Mutations have not previously been seen at VP2 position 556, which is located along the two-fold axis (Figure 3B–C), but we observed this same mutation in all viruses passaged in gray fox cells, strongly suggesting that the 556-Asn change was associated with gray fox infection. As we did not detect any parvoviral DNA in gray foxes (Table 1), we cannot correlate these findings to viruses in nature. Rac3 already contained an Asn mutation at VP2 position 323 (Table 2), one of the two mutations (i.e., VP2 positions 93 and 323) that experimentally allowed FPV to be able to infect dog cells [9]. However, because Rac3 is an FPV-like virus, it did not replicate efficiently in dogs cells and was not detected beyond the fifth passage. The other known canine adaptive mutation (position 93) was not detected. Additionally, multiple passages of Rac3 in both A72 cells and another dog cell line (MDCK), where 24 attempts were made to infect each cell line, did not result in Rac3 adapting to dog cells.

In marked contrast, pandemic CPV-d (CPV-2) underwent extensive evolution during passage, resulting in 18 mutations at nine different sites in VP2 (Figures 2A and 3D–F). Four of these mutations – at VP2 positions 93, 103, 375, and 562 – were observed after passage in more than one carnivore cell line, suggesting that they were important for host adaptation. For example, Ala103 to Val was observed during ferret, gray fox, cat, and raccoon cell passage, while the Asn to Asp mutation at position 375 occurred in all hosts except the dog, showing that parallel selection of host adaptive changes can occur frequently. Many of these parallel mutations have previously been observed in field isolates recovered from a variety hosts in nature (Figure 2B–C) [9,12,13,32–34], further supporting their importance in determining host range. In contrast, some mutations were only observed in one particular host passage series (e.g., VP2 residues 29 in gray fox and 40 in mink; Figure 2A). Such residues were not exposed on the surface of the capsid (Figure 3D–F), suggesting that they were not involved in specific TfR interactions.

Previous mutational studies with FPV have shown that VP2 residues 93 and 323 control the interaction with the canine TfR and infection of domestic dog cells [9]. Additionally, changing residue 103-Ala in CPV-2 to the FPV sequence of 103-Val greatly decreased replication in dog cells [9], suggesting that residues 93, 103, and 323 were important host range mutations needed for dog adaptation. The importance of position 103, located in a pocket underneath the 300 loop (Figure 3F), in specific host cell adaptation was verified during experimental evolution studies, as that residue changed from an Ala to a Val in all hosts except for dog and mink (Figure 2C). The absence of the mutation to 103-Val in mink cells suggests that similarities may exist between the dog and mink TfRs in virus binding, and this was supported by the observation that passage of CPV-like raccoon viruses in dog and mink cells selected the same 300 Asp to Gly change (Figure 2C), characteristic of all dog viruses in nature (Table 2) [35]. However, passage of CPV-2 in mink (as well as ferret) cells resulted in a change to FPV-like residues at VP2 positions 93 (Asn to Lys) and 375 (Asn to Asp) (Figures 2 and 3), which have been implicated in TfR binding or hemagglutination, respectively [9,19,32,36–38], demonstrating that the specific host-virus interactions are likely to be complex.

As passage of CPV-2 in the various hosts resulted in multiple mutations, we wanted to examine how such mutations may be affecting the fitness of the passaged viruses relative to their cognate non-passaged stock virus. To test this, we chose passage 0 (stock virus) and passage 20 (passaged in Mpf cells) CPV-2. This particular virus-cell combination was chosen because CPV-2 had multiple mutations occurring at key host range positions (VP2 93, 103, 375, 562) after 20 passages in Mpf cells, suggesting it would be a good candidate for altered infectivity, and Mpf cells were shown to be highly susceptible to parvoviruses, and thus amenable to detecting possible differences in the magnitude and duration of virus production. Single (multi-step) growth curve analysis between passage 0 and passage 20 CPV-2 demonstrated that the two viruses had similar growth curves up to day post-infection (DPI) 2 (Figure 4A). However, the infectious titer of the passage 20 virus began to rise dramatically (relative to the stock virus) starting at DPI 3, suggesting an increase in virus binding and/or entry associated with the observed mutations. By DPI 4, obvious differences in cytopathology were evident between the two viruses (Figure 4B), most likely a consequence of the 32-fold mean increase in titer in the passage 20 virus (6.38×10^6 $TCID_{50}$/mL) relative to passage 0 virus (1.97×10^5 $TCID_{50}$/mL) at that time point.

To further investigate these differences, we performed competition assays, again using passage 0 and passage 20 (in Mpf cells) CPV-2. Similar to the single growth curve analysis, competition of the two viruses in Mpf cells at a 1:1 ratio resulted in approximately equal amounts of virus (based on Sanger sequencing chromatogram peaks) at DPI 2, followed by the passage 20 CPV-2 clearly outcompeting the stock virus by DPI 4 (Figure 4C). Remarkably, when competition experiments were conducted using a 10:1 ratio in favor of the passage 0 virus, this stock virus was the main virus present at DPI 2, but was outcompeted by the passage 20 virus by DPI 4 (Figure 4C), demonstrating that the passage 20 virus had acquired considerable fitness advantages over its progenitor virus. Identical results were obtained when analyzing another key adaptive mutation (VP2 375).

Many CPV-like viruses isolated from raccoons do not efficiently bind the dog TfR or infect dog cells [29]. However, we observed that the prototype raccoon CPV strain (Rac118) quickly and repeatedly gained the dog host range after passage in A72 cells through a mutation at VP2 codon 300, converting Asp (GAT) to a Gly (GGT) (Figure 5 A–B), and that mutation was also observed during passage in MDCK cells. This suggests that either the 300-Gly variant was present at an undetectable level (by Sanger sequencing) during the stock preparation in NLFK cells, or was present in the original raccoon tissue samples of that isolate. To determine if mutations at position 300 during canid cell passage were common to other similar host-derived viruses, another raccoon virus, Rac334 (see Figure 1 and Table 2), was passaged *in vitro*. Rac334 also varied at position 300 during passage in A72 cells, but in this case exhibiting an Asp to a Val (GAT to GTT) mutation, which also introduced the dog host range. Rac334 also mutated to a 300-Val in gray fox cells. Hence, this analysis demonstrates that CPV-like raccoon viruses can adapt to infect canid cells through multiple mutational pathways.

VP2 residue 300, along with the adjacent residues, appears to be central to dictating host range [11,35,36]. Several additional mutations at position 300 were observed in the raccoon viruses when passaged in alternate carnivore cells. Rac118 (and Rac334) showed a 300-Asp to His substitution during ferret cell infection (Figure 5C), and that mutation became fixed by passage 5. By passages 10–20, the neighboring residue 301 was polymorphic for

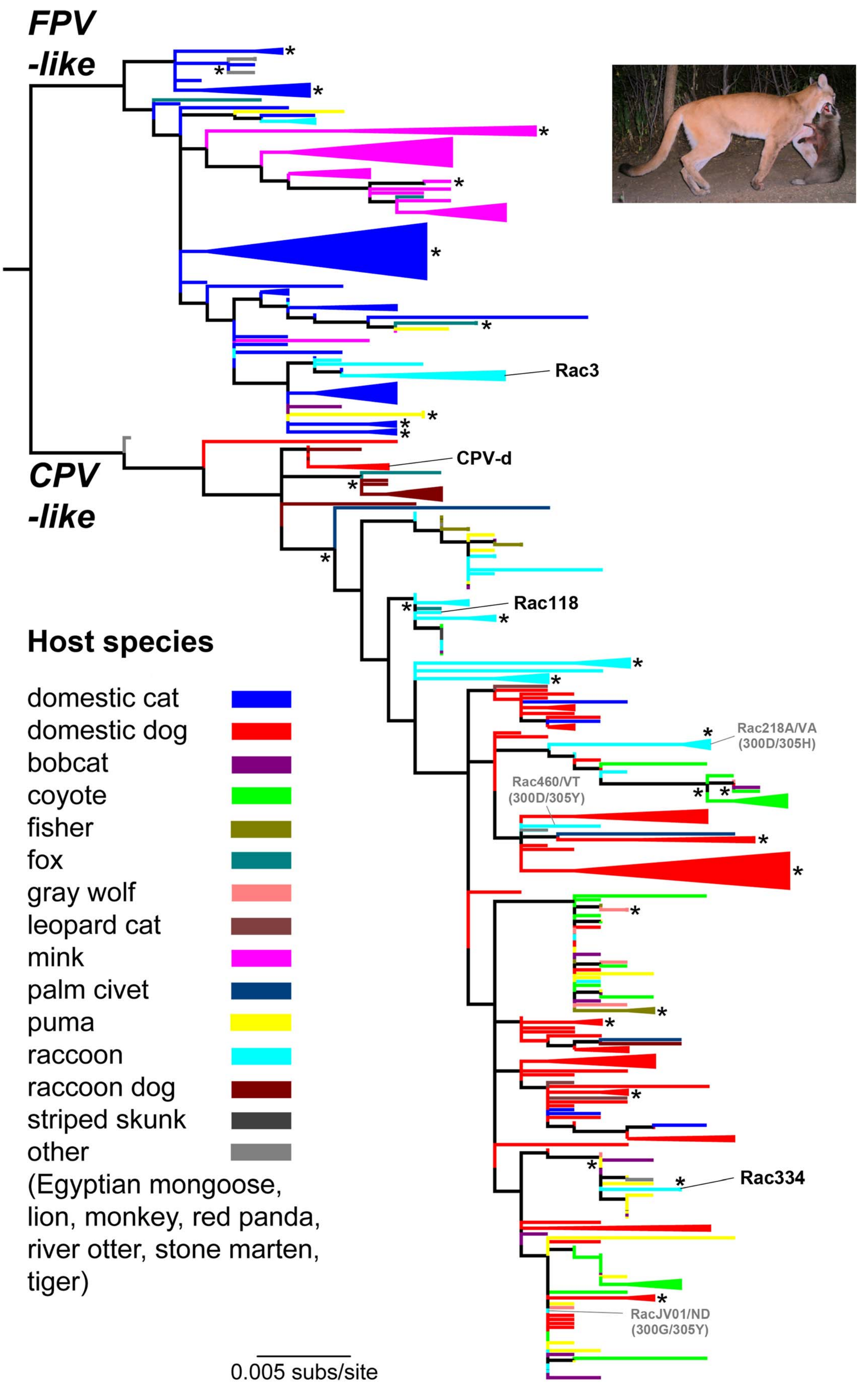
FPV
-like
CPV
-like
Rac3
CPV-d
Rac118
Rac218A/VA
(300D/305H)
Rac460/VT
(300D/305Y)
Rac334
RacJV01/ND
(300G/305Y)
Host species
domestic cat
domestic dog
bobcat
coyote
fisher
fox
gray wolf
leopard cat
mink
palm civet
puma
raccoon
raccoon dog
striped skunk
other
(Egyptian mongoose,
lion, monkey, red panda,
river otter, stone marten,
tiger)
0.005 subs/site

Figure 1. Phylogenetic relationships of 343 VP2 capsid protein nucleotide sequences of parvoviruses recovered from wild and domestic carnivores. Branches are color-coded by host species (see key; no differentiation is made for the small number of viruses sampled from fox species of the genus *Vulpes*). Monophyletic groups comprising viruses sampled from a single host are 'collapsed' and shown as triangles of the appropriate color. The viruses used in the adaptation studies [FPV/Raccoon/TX/Rac3/78 (Rac3), CPV-2/Dog/NY/CPV-d/79 (CPV-d), CPV/Raccoon/VA/118-A/07 (Rac118) and CPV/Raccoon/334-A/CA/10 (Rac334)] are highlighted to reveal their origin. Select examples of cross-species transfers between raccoons and dogs or dog-like canids are shown in gray; see text for details. The tree is rooted using the molecular clock based scheme determined previously [28] and all horizontal branches are drawn to a scale of nucleotide substitutions per site. Nodes with bootstrap values >75% are marked by an asterisk (*) symbol, and for ease of representation are sometimes shown to the right of the relevant nodes. Inset: Tentative novel route of cross-species parvovirus transmission among wild carnivores. Based on phylogenetic analysis and the field evidence shown here with puma carnivory on raccoons, predation and/or scavenging of infected animals may provide an alternate pathway for cross-species parvovirus transmission, in addition to the prototypical fecal-oral route found in domestic systems. Picture courtesy of Ashley Gramza, Colorado State University.
doi:10.1371/journal.ppat.1004475.g001

both Thr and Ala (Figures 5C–D). This finding (in both viruses) suggests that once the 300-His became fixed during ferret passage, the accompanying, but subsequent, change at position 301 arose due to either steric hindrance between the newly selected residue at position 300 and the original 301 residue (e.g., 300-His and 301-Thr) (Figure 5C), or because the 301 change compensated for the altered receptor interaction with the ferret TfR once the change at position 300 occurred. The detection of the 301-Ala mutation in association with alternate position 300 residues in nature, such as in the 300-Ser in masked palm civets (*Paguma larvata*) (Table 2), supports this hypothesis. The identification of changes at VP2 positions 300 and/or 301 in the raccoon viruses during passage in dog, cat, ferret, and mink cells provides additional support for a major role of this capsid region in host adaptation.

To further investigate how changes at position 300 may influence infection, we performed relative infectivity assays of passage 0 and passage 20 Rac118 (passaged in A72 cells) in various hosts including the domestic cat, dog, and ferret (Figure 5E). Infection of cat (NLFK) cells with non-passaged or passage 20 Rac118 resulted in similar numbers of infected cells, suggesting that cat cells are equally permissive to viruses containing both 300-Asp (passage 0) and 300-Gly (passage 20), consistent with what is observed in nature (i.e., felid species are susceptible to both raccoon and dog CPV isolates). However, both dog (A72) and ferret (Mpf) cells were highly refractory to non-passaged Rac118. In the case of dog cells, selection of the 300-Gly results in this host range barrier being overcome, as evident in the high levels of cytopathic effects and infection of the remaining cells at DPI 6 of the passage 20 virus (Figure 5E). Interestingly, passage of Rac118 in dog cells also results in substantial increases in infectivity in ferret cells (Figure 5E) (which is normally associated with a 300-His during infection with Rac118 rather than the 300-Gly), demonstrating that adaptation of the raccoon virus to one host (dog) may inadvertently lead to a virus more fit in an additional host (in this example, ferret).

TfR host-specific sequence variation and evolution

To obtain a preliminary understanding of the host involvement in the selection of the capsid mutations we observed, we sequenced the cDNA of the TfRs from the six hosts used in the experimental evolution studies. Alignments of the full-length TfRs demonstrated amino acid identity from 98% among closely related species (mink and ferret, both mustelids) to 88% (domestic cat versus all caniform TfRs) (Figure S1). Overall, there were 146 variant residues (19.0%) in the TfR among the six hosts (Figure 6 and Figure S1), such that carnivore parvoviruses must overcome a considerable amount of diversity to be able to infect a wide range of different hosts. Previously, we have demonstrated that the parvovirus capsid interacts with the apical domain of the TfR to infect domestic cat and dog cells and that a number of residues in that domain, such as 221/222 Leu, influence virus binding [18,20,21]. The TfR apical domain sequences of the six hosts showed 40 variable residues (Figure S2), with the majority of these changes located on the top and side of that domain (Figure 6B–C), including a number of positions (207, 216, 218, 301, 304, 379, 381) that have been previously suggested to be under positive selection [20]. Other domains also contained frequent variable sites, including the side portion of the protease domain (encompassing residues 582–588) that underlies the apical domain (Figure 6C), although it is currently not known if and/or how these sites influence the pattern of capsid mutations in the virus.

We have previously shown that only a small group within the Canidae (coyote and domestic dog) contain an Asn at TfR residue 384 that introduced a new glycosylation site which blocked FPV infection [18–20], suggesting this barrier to infection was limited only to canids of the domestic dog lineage (see Figure S2). Consequently, along with the gray fox, we sequenced the cDNA of the TfRs from the gray wolf and Arctic fox, all members of the Canidae family. The 384 glycosylation site (NLT) was only present in the gray wolf TfR, demonstrating that wolves, coyotes and domestic dogs, and not the fox species, are the only carnivores known to contain this additional glycan. Additionally, the gray wolf, coyote, and domestic dog (A72) possessed identical TfR sequences (Table S2).

Discussion

Natural host-specific variation of parvoviruses

To explore the relationship between virus mutations and host range, we compared the natural variation of parvoviruses recovered from different carnivore hosts and the results of experimental studies involving extended cell culture passage of viruses. The central aims were to define how barriers to infection may be overcome and the mutational pathways by which viruses with altered host ranges may arise, and to identify parallel, and hence likely adaptive, mutations in both nature and *in vitro*. There are over 280 recognized species in the mammalian order Carnivora [39], and it is unclear how many of these act as natural reservoirs for viruses related to FPV and CPV. While periodic infections of wild carnivores have been known for many years [22–24,40–42], our analysis demonstrates that parvoviruses are much more widespread in North American carnivore species than previously recognized (Figure 1 and Table 1), and this may also be the case among carnivores in other geographic regions. Although it is now evident that many different sylvatic cycles involving various carnivore species operate in nature, the direction of transfer between domestic and wildlife species and among different wild carnivores is often unclear. However, our phylogenetic analysis suggests that this is likely to be both relatively common and bi-directional, with ongoing transmission occurring back and forth between different hosts. In addition, almost all of the viruses that we detected appear to be CPV-like, suggesting that CPV is

Table 2. Amino acid mutations in the VP2 capsid protein of a representative set of parvoviruses including the prototypes of FPV, CPV-2, and CPV-2a, and select CPV host range variants, highlighting the polymorphic nature of residue 300.

		VP2 residue															
**Virus**	**Host**	**80**	**87**	**93**	**101**	**103**	**224**	**232**	**297**	**300**	**301**	**305**	**323**	**375**	**426**	**564**	**568**
FPV prototype	Domestic cat	K	M	K	T	V	G	V	S	A	T	D	D	D	N	N	A
Rac3/TX/78 (FPV-like)[a]	Raccoon	K	M	K	T	V	G	V	S	A	T	D	N	D	N	N	A
CPV-d/NY/79 (CPV-2 prototype)[a]	Domestic dog	R	M	N	I	A	G	I	S	A	T	D	N	N	N	S	G
CPV-2a prototype	Domestic dog	R	L	N	T	A	G	I	S/A	G	T	Y	N	D	N	S	G
RacJV01/ND/13 (CPV-2a)	Raccoon	R	L	N	T	A	G	I	A	G	T	Y	N	D	N	S	G
Rac460/VT/12 (CPV-2a-like)	Raccoon	R	L	N	T	A	G	I	A	D	T	Y	N	D	N	S	G
Rac118/VA/07 (CPV-2a-like)[a]	Raccoon	R	L	N	T	A	R	I	A	D	T	D	N	D	N	S	G
Rac37/WI/10 (CPV-2a-like)	Raccoon	R	L	N	T	A	G	T	S	D	T	D	N	D	N	S	G
Rac334/CA/10 (CPV-2a-like)[a]	Raccoon	R	L	N	T	A	G	I	A	D	T	H	N	D	D	S	G
Rac218A/VA/11(CPV-2a-like)	Raccoon	R	L	N	T	A	G	I	A	D	T	H	N	D	N	S	G
RPPV/China/04 (CPV-2a-like)	Red panda	R	L	N	T	A	G	I	A	V	T	Y	N	D	N	S	G
MCPV/China/08 (CPV-2a-like)	Palm civet	R	L	N	T	A	G	I	S	S	A	Y	N	D	N	S	G

[a]Viruses used in the experimental evolution studies herein.

doi:10.1371/journal.ppat.1004475.t002

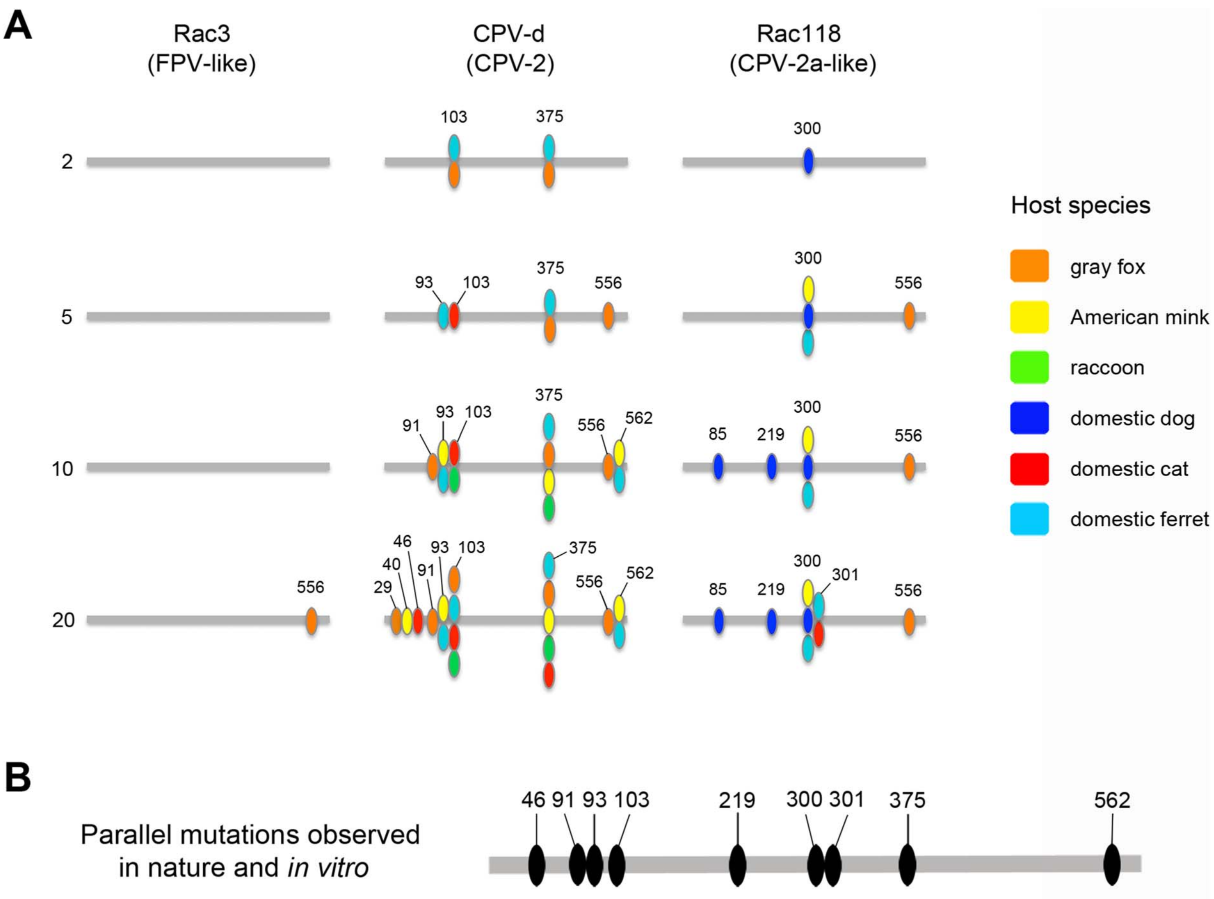

C

VP2 position	Mutation	*In vitro* host(s)	Virus	Mutation	Natural host(s)	Virus	Function [reference]
29	G → S		CPV-d	*	*	*	Unknown
40	I → V		CPV-d	*	*	*	Unknown
46	N → D		CPV-d	N → S	masked palm civet	CPV-2a	Unknown
85	N → I		Rac118	*	*	*	Unknown
91	A → T		CPV-d	A → T	puma	CPV-2a	Adjacent to VP2 93; see Figure 3D-F
93	N → K		CPV-d	K → N	domestic dog[a]	CPV-2	TfR binding/host range [9,12,13]
103	A → V		CPV-d	V → A	domestic dog[a]	CPV-2	Infectivity/host range [9]
219	I → M		Rac118	I → V	raccoon dog	CPV-2	Unknown
300	D → G		Rac118	G → D	raccoon, leopard cat	CPV-2a	TfR binding/host range [29,34-36]
	D → H		Rac118	*	*	*	TfR binding/host range [29,34-36]
301	T → A		Rac118	T → A	masked palm civet	CPV-2a	TfR binding/host range [11,36]
	T → I		Rac118	*	*	*	TfR binding/host range [11,36]
375	N → D		CPV-d	D → N	domestic dog[a]	CPV-2	Hemagglutination/calcium binding [9,32,37]
556	D → N		Rac3, CPV-2, Rac118	*	*	*	Unknown
562	V → L		CPV-d	V → L	puma, American mink	FPV	Under positive selection [33]
				V → L	raccoon dog	CPV-2	Adjacent to VP2 564 and 568 [12,38]

[a]Presuming a FPV-like origin of CPV-2 (see Table 2)

Figure 2. Mutations in the VP2 capsid protein sequences of parvoviruses observed during the experimental evolution studies and a comparison to those found in nature. (A) Amino acid changes occurring in the VP2 capsid protein of parvoviruses after cell culture passage. Viruses were passaged for 20 weeks in domestic dog (A72), domestic cat (NLFK), domestic ferret (Mpf), raccoon (Pl1Ut), gray fox (FoLu), and American mink (Mv1Lu) cells and cultures were collected at passages 2, 5, 10, and 20 to determine the mutations that occurred. Gray bars indicate the linear primary sequence of VP2 from residues 1–584 and passage series is indicated on the far left. Each mutation arising during passage is highlighted by its residue number (not to scale due to space limitations in some instances) and is colored-coded according to the carnivore species as indicated by the key. (B) Amino acid positions in VP2 observed to have mutations both in viruses recovered from nature and also in those passaged under experimental conditions, suggesting they are of adaptive importance. (C) Comparison between mutations derived from cell culture passage and those found in nature, indicating the mutation observed and the hosts involved. The possible or known functional importance of such mutations on host infection is indicated, along with appropriate references.

doi:10.1371/journal.ppat.1004475.g002

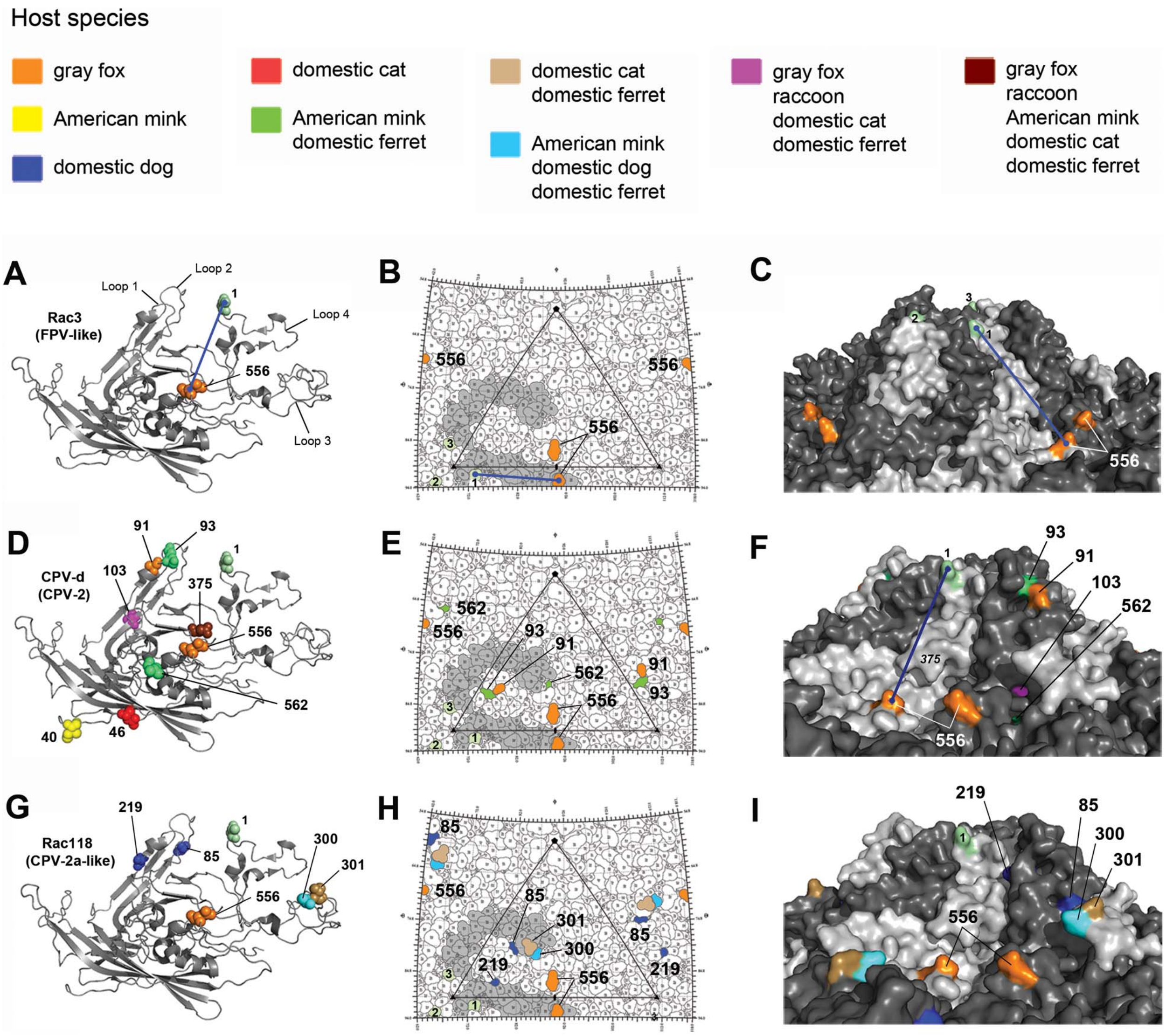

Figure 3. Structural location of the VP2 capsid mutations arising during experimental evolution studies in the six different hosts. The three-dimensional ribbon structure of a single VP2 monomer of the parvovirus capsid is shown in panels A, D, and G, with residues that mutated during cell culture passage highlighted by spheres which are color-coded according to the host species in which they arose (see key). Note that residue 440 (denoted as a lime green '1') is not a mutation and is shown only for orientation between the left, middle, and right panels and to highlight the top of the three-fold spike. A blue line stretching between residue 440 and 556 is shown in panels A, B, C, and F, again for orientation between panels. Note that some residues seen in panels A, D, and G are not present in the middle and right panels, as they are not directly surface exposed. Similarly, residue 375 shown in panel D is hidden near the surface and its underlying location is highlighted by italics in panel F. Also note that the VP2 crystal structure lacks residues 1–36, and thus the mutation at position 29 in CPV-d (Figure 2) is not shown. The stereographic road map of surface-exposed VP2 residues is shown in panels B, E, and H, with an icosahedral asymmetric unit of the capsid highlighted with a triangle [36]. The single monomer shown in panels A, D, and G is highlighted in grey in each roadmap. The surface rendition of the CPV capsid is shown in panels C, F, and I. Note that in panel C, the three 440 residues (numbered 1–3) from the three different VP2 monomers that constitute the three-fold spike are visible and are equivalent to those shown in panel B.
doi:10.1371/journal.ppat.1004475.g003

displacing FPV in the wild, although this will need to be confirmed through ongoing surveillance studies.

In vitro passage selects for host-specific changes in viruses

To better understand the molecular changes associated with the natural variation of the carnivore parvoviruses, we performed extended cell culture passage with a number of parvovirus isolates from different hosts that are representative of the evolutionary history of the group. These included (i) the original prototype pandemic CPV-2 (CPV-d) that circulated globally in dogs for a very short time (1978 to 1979) before being displaced by CPV-2a, (ii) an FPV-like isolate from a raccoon (Rac3) that possessed an important canine adaptive mutation (VP2 323-Asn), as well as (iii) the prototype CPV-like raccoon virus (Rac118) isolated in 2007 (Table 2). After cell culture passage, these viruses differed dramatically in the mutations that arose in the different carnivore hosts (Figures 2 and 3). In particular, the FPV-like strain (Rac3) showed little or no adaptive changes in VP2 over 20 passages in six different cell lines, suggesting that it was broadly able to infect a

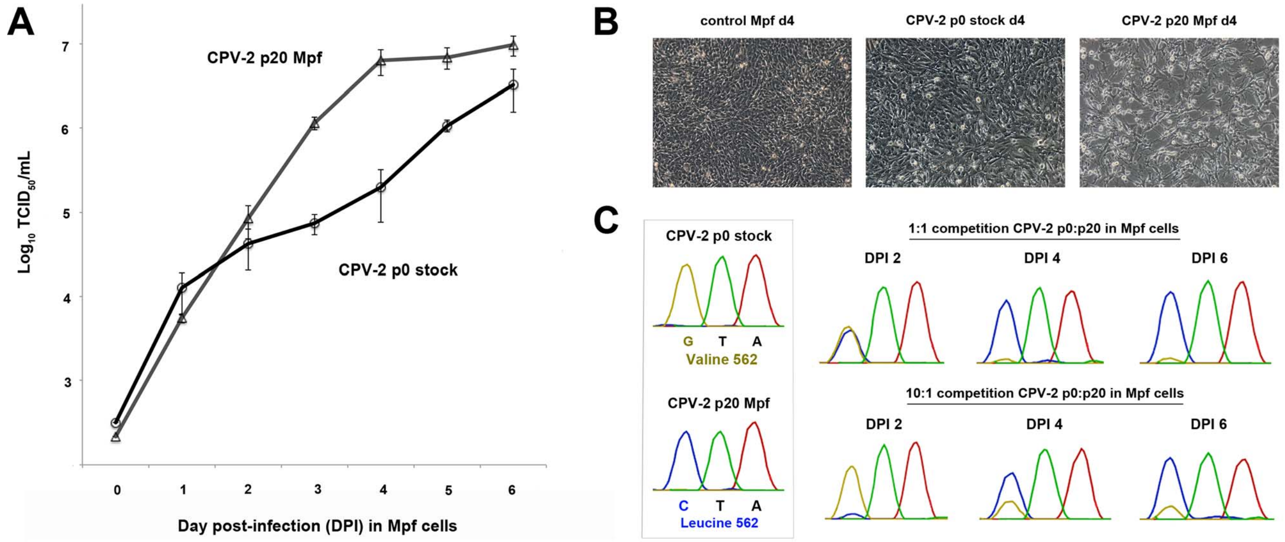

Figure 4. Multi-step single growth curve analysis and competition assays between non-passaged and terminally passaged viruses to detect changes in fitness. (A) Single growth curve analysis of ferret (Mpf) cells infected with passage 0 (p0 stock) CPV-2 or CPV-2 passaged 20 times in Mpf cells (p20 Mpf) over six days. Data shown are from experiments performed in triplicate with error bars indicating standard deviations. (B) Phase contrast images of control, p0 CPV-2-infected, and p20 CPV-2-infected, ferret cells at day post-infection (DPI) 4. Note the increased cytopathology of Mpf cells infected with the passage 20 virus in association with the 32-fold relative increase in titer over stock virus observed at DPI 4 in panel A. (C) Competition assays between p0 stock and p20 Mpf CPV-2 in ferret cells. VP2 residue 562, which is a valine (GTA) in the p0 stock and a leucine (CTA) in the p20 virus (see Figure 3E–F for location on the capsid), is one of the key adaptive mutations that differentiates the two viruses and was chosen as a marker to measure changes in virus composition over time. Chromatograms of residue 562 at DPI 2, 4, and 6 are shown for competition assays at either a 1:1 or a 10:1 ratio of p0:p20 virus. Note that the passage 20 virus outcompetes the original stock virus at both the 1:1 and 10:1 ratios.
doi:10.1371/journal.ppat.1004475.g004

wide range of feliform and caniform hosts without mutation (Figure 2; see Tables 1 and S2 for suborder designations). In contrast, CPV-2 underwent extensive mutation as it was passaged in culture, with 18 capsid mutations at nine sites amongst the six host species tested (Figures 2 and 3). However, no mutations arose during passage in dog cells, suggesting the virus is well-adapted to dogs (and likely to closely related canids such as wolves and coyotes), while VP2 mutations are required to allow CPV-2 to efficiently infect other carnivore hosts. Remarkably, although Rac3 and CPV-2 differed dramatically in how they adapted to various carnivore hosts, they differ by only nine amino acids in their capsid proteins (Table 2) and share >99% nucleotide identity across their genomes, and thus would be considered to be minor variants of the same strain in most virus systems. These results therefore clearly demonstrate that many different aspects of the biology and ecology of parvoviruses can influence host adaptation, including their evolutionary and phylogenetic background, the duration of time they have been naturally circulating, as well as their degree of host restriction and/or specialization (i.e., normal host range) [43].

To determine how the observed mutations seen during passage of CPV in various hosts may be affecting fitness levels, non-passaged and terminally passaged viruses were tested by single growth curve, competition, and relative infectivity assays. Remarkably, some passaged viruses such as ferret-passaged CPV-2 had large gains in fitness relative to their progenitor virus, such that it could outcompete non-passaged virus even when starting at a 10-fold lower concentration (Figure 4C). These results demonstrate that even apparently subtle capsid mutations occurring during host passage may have considerable effects on the efficiency of infection. Another key finding was the repeated mutation at VP2 position 300, which lies within a capsid region that controls binding to the TfR and subsequent infection [36], during passage of CPV-derived raccoon viruses. VP2 position 300 is also highly polymorphic in nature [29,34,44] (Table 2) and had a remarkable degree of parallel evolution in our phylogenetic analysis. As domestic dogs have been shown to be refractory to infection with CPV-derived raccoon viruses [29], a key question is how these viruses transverse such a barrier. Here we demonstrate that the block to canine infection was overcome through a single VP2 mutation (300-Asp to Gly or Val) (Figure 5E), showing that the species barrier can be traversed through multiple mutational pathways. This adaptive flexibility suggests that the host barrier to dogs and possibly other canid species could be overcome relatively easily in nature, as other commonly observed mutations in raccoon CPV isolates appear not to be required (e.g., VP2 residues 224, 232, 297, 305; see Table 2). Differences in VP2 codon 300 were also observed after transfer of dog or dog-like CPVs to raccoons in nature (e.g., Rac460/VT/12; Figure 1 and Table 2). If raccoons acted as an important host in the evolution of CPV-2a as previously suggested [29], these results indicate that only a single mutation (VP2 position 300) was needed for raccoon-adapted viruses to be transferred to dogs or dog-like canids.

Emergence of CPV as a new pandemic pathogen

An outstanding question that remains to be fully explained is how pandemic CPV-2 emerged and from which hosts? Although it is often presumed that CPV-2 emerged from an FPV-like virus of domestic cats, the widespread circulation of parvoviruses among diverse carnivore hosts suggests that this may not be the case [28]. Some additional clues are provided by the experimental evolution studies of CPV-2 shown here, where we observed mutations identical to those observed in FPV. Two VP2 mutations (Lys93Asn and Asp323Asn) are central to allowing FPV to become infectious to dog cells [9], and passaging CPV-2 in two

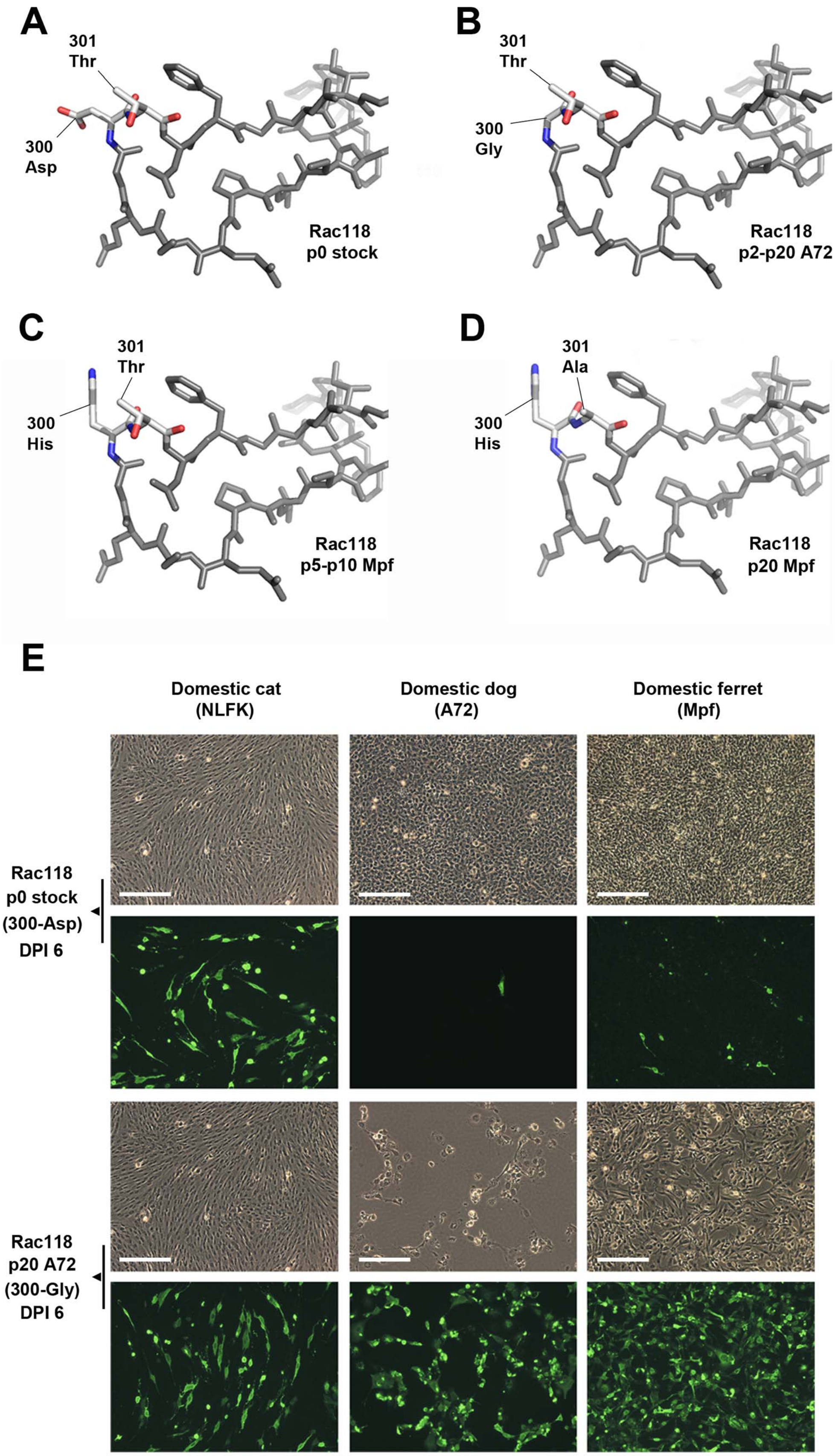
A
301
Thr
300
Asp
Rac118
p0 stock
B
301
Thr
300
Gly
Rac118
p2-p20 A72
C
301
Thr
300
His
Rac118
p5-p10 Mpf
D
301
Ala
300
His
Rac118
p20 Mpf
E
Domestic cat
(NLFK)
Domestic dog
(A72)
Domestic ferret
(Mpf)
Rac118
p0 stock
(300-Asp)
DPI 6
Rac118
p20 A72
(300-Gly)
DPI 6

Figure 5. Mutations in the VP2 300 region of CPV that are involved in receptor binding and host range expansion. (A) Stick representation of the 300 loop region of the prototype raccoon CPV (Rac118), demonstrating the raccoon-specific 300-Asp, along with 301-Thr, a residue common to all parvovirus isolates. (B) 300 loop region of Rac118 after passage in dog cells, resulting in the selection of the 300-Gly, a prerequisite mutation for cross-species transfer of raccoon viruses into dogs. (C) 300 loop region of Rac118 after passage in ferret cells, resulting in a 300-His mutation, which is invariably followed by a (D) 301-Thr to -Ala change. (E) Relative infectivity of domestic cat, dog, and ferret cells to non-passaged Rac118 (p0 stock) and Rac118 after 20 passages in A72 cells (p20 A72). Upper panels show phase contrast images of cells on day post-infection (DPI) 6, while lower panels show viral antigen detected using a rabbit anti-CPV VP1/VP2 antibody and an Alexa Fluor 488 goat anti-rabbit IgG (note phase contrast and fluorescence images are not overlays). Also note that while cat cells are equally susceptible to either virus, only the A72 passage 20 Rac118 (containing a 300-Gly) is highly infectious to dog and ferret cells and also induces cytopathic effects, most notably in dog cells (thus limiting the amount of observed fluorescence in that particular case). Scale bar = 200 µm.
doi:10.1371/journal.ppat.1004475.g005

mustelid species (mink and ferret) consistently resulted in a change of Asn-93 to Lys as seen in FPV (Figure 2). Such viruses would resemble intermediates between FPV and CPV-2, and indeed such intermediate-like viruses have been reported in wild red foxes (*Vulpes vulpes*), leading to the suggestion that a fox parvovirus was the direct ancestor of CPV-2 [45]. However, as the same VP2 mutations that occur in wild foxes in nature (e.g., 103-Val) can be observed by passage of CPV-2 in fox cells (Figure 2), such isolates may well be CPV-2 mutants that are selected during fox infection, thereby demonstrating the importance of accounting for the directionality of mutations.

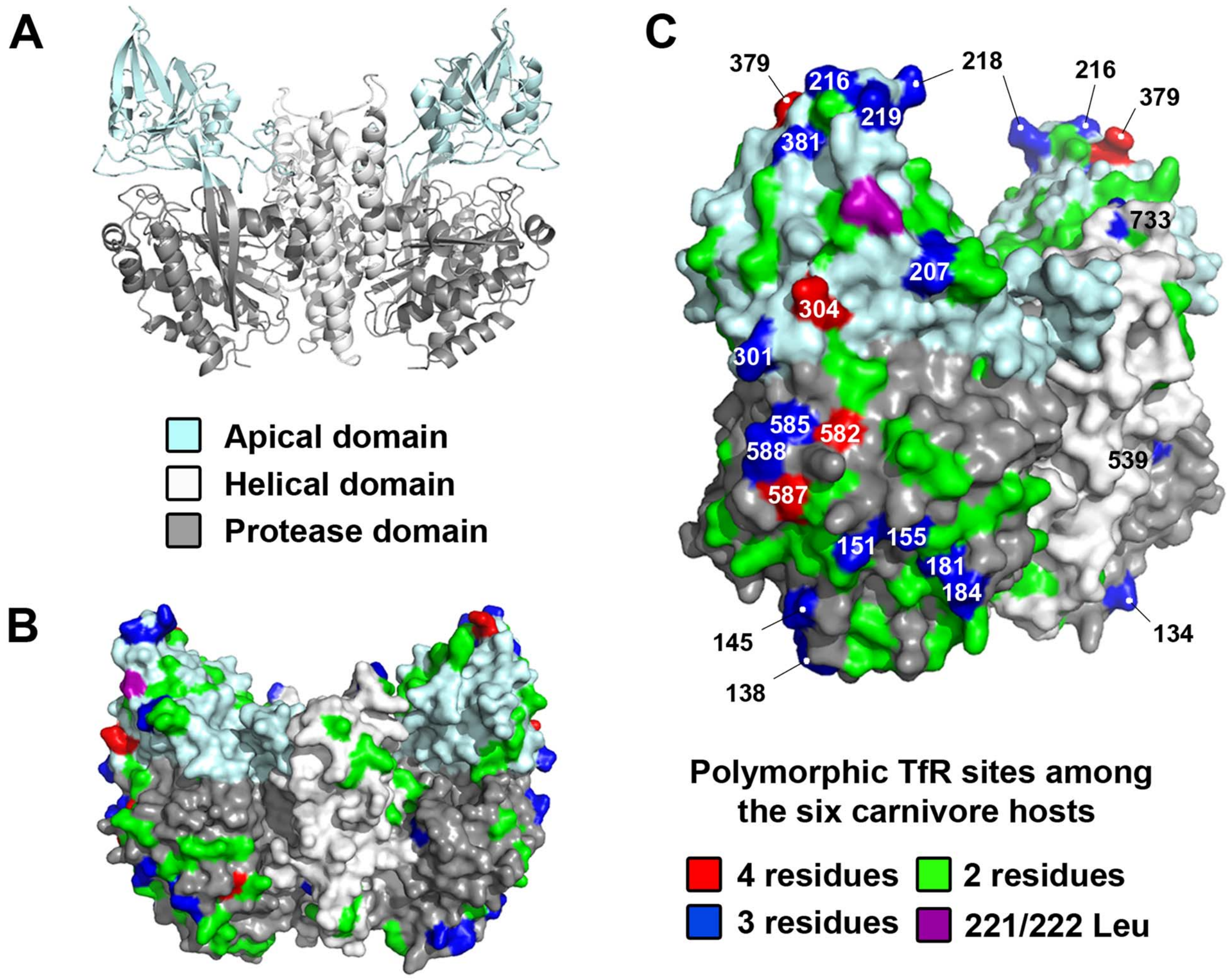

Figure 6. Structural location of mutations in the TfR among the six carnivore hosts analyzed in the experimental evolution studies. (A) Ribbon diagram of the crystal structure of the human TfR homodimer [17], with the three individual domains within the ectodomain color-coded. (B) Structural mapping of all the variable sites in the dog, cat, ferret, raccoon, gray fox, and American mink TfRs on the surface rendition of the human TfR shown from a front view. (C) The same model as in B shown in a counterclockwise ~45° rotation to highlight the number of variable sites (including those under positive selection) found along the edge and top of the receptor. Variable sites are color-coded according to the number of different amino acids found at those positions, with amino acid sites that contain 3 or 4 different residues numbered. TfR Leu 221 (felid) or 222 (canid) is a residue that has been previously shown to be critical in parvovirus binding [21].
doi:10.1371/journal.ppat.1004475.g006

TfR sequence and structure selects virus variation

Many of the mutations selected during passage in cells from different hosts fell within the known receptor-binding regions of the capsid surface (Figure 3) [9,11,18,19,21,36] and we expect that these mutations influence receptor binding to give more efficient cell infection as suggested by the growth curve and competition assays (Figure 4), which will be examined further in future studies. In some cases, more than one mutation in a single virus was selected during host cell passage, and these mutations may act together to influence tropism. Sequential selection of mutations may allow compensation for certain changes, as seen for the VP2 301 change following the 300 mutation (Figure 5C–D). Additionally, many parallel mutations in regions of the capsid known to influence host range were observed after passage in cells of related species (e.g., mustelids, such as mink and ferret; VP2 93 and 562) that show a high level of sequence similarity (98%) in their TfRs, further suggesting that VP2 interactions with the host receptor structures selected the mutations. Moreover, viruses isolated from carnivores with identical TfRs (gray wolf, coyote, domestic dog) were remarkably similar in their VP2 sequences and showed frequent parallel mutations, again supporting a key role of the TfR in the observed VP2 changes.

Understanding host tropism and pathways of virus emergence

While the emergence of CPV as a pathogen has been of great interest due to the dramatic nature of its pandemic spread during 1978, we demonstrate here that more subtle changes in the receptor-binding structures of the viral capsid are also important for infection of many other hosts. Although the details of the evolutionary pathways that allowed pandemic CPV-2 and CPV-2a to emerge are still unclear, we have shown that capsid mutations seen in pandemic viruses can readily be recapitulated *in vitro* in different carnivore hosts and that these changes affect virus fitness. Our surveillance results force us to re-examine the pathways of parvovirus emergence by showing that many wild carnivore species have likely been important, yet unappreciated, hosts during CPV evolution. Additionally, we demonstrated that some of these parvoviruses may cross species barriers through single mutations that allow them to infect previously non-susceptible hosts, and that differences in the genetic background and evolutionary histories of very similar viruses can profoundly influence their ability to adapt to and infect a new host. Overall, these findings provide new insights into the mechanisms that dictate parvovirus host switching and, ultimately, virus emergence.

Materials and Methods

Ethics statement

Wild carnivores sampled in this study were collected by two agencies: (i) United States Department of Agriculture-Animal and Plant Health Inspection Service-Wildlife Services (USDA-APHIS-WS) and the (ii) North Dakota Game and Fish Department. The corresponding author (ABA) was not involved in the collection or trapping of carnivores for this study. Animals were randomly sampled in conjunction with ongoing state and federal surveillance and/or nuisance/damage control programs. The primary statutory legislation for USDA-APHIS-WS is the Act of March 2, 1931 (46 Stat. 1468; 7 U.S.C. 426-426b) as amended, and the Rural Development, Agriculture, and Related Agencies Appropriations Act of 1988 (Public Law 100–202, 7 U.S.C. 426c), which provides legal authority for the control of wildlife diseases and nuisance wild mammals and birds in the United States. All animals tested from the state of North Dakota were collected in accordance with state law as outlined in North Dakota State Century Code 20.1–07 and 20.1–08 and through the Governor's Furbearer Proclamations for the years 2010–2014. Methods of take of furbearer species that are approved in the state of North Dakota are managed by the North Dakota Game and Fish Department and were developed and approved as humane through a National Best Management Practices program in conjunction with an international program administered by the Association of Fish and Wildlife Agencies (AFWA).

Phylogenetic analysis of carnivore parvoviruses

New parvoviruses from wild carnivores were detected from tissue as described previously [28]. Briefly, DNA was extracted from spleen, gastrointestinal tract, and/or tongue tissue (0.3 cm^3) using an E.Z.N.A. Tissue DNA kit (Omega Bio-tek, Norcross, GA) and then screened for parvovirus using primers amplifying a short stretch (639 nt) of VP2 (primers available from authors upon request). All positive samples were verified by sequence analysis. Select samples that were positive on the initial screen were further amplified to sequence the entire VP2 gene (1755 nt). Due to the recognition of mutations arising during cell culture passage, only VP2 sequences that can be amplified directly from tissue should be used for phylogenetic analysis. New complete VP2 sequences obtained from this study have been submitted to GenBank under the accession numbers KJ813827-KJ813895 (Table S1).

In total, we obtained 68 additional full-length VP2 sequences from the following eight host species: coyote (*Canis latrans*), mountain lion or puma (*Felis concolor*), bobcat (*Lynx rufus*), fisher (*Martes pennanti*), raccoon (*Procyon lotor*), gray wolf (*Canis lupus*), North American river otter (*Lontra canadensis*), and red fox (*Vulpes vulpes*) (Table S1). These 68 sequences were combined with 275 complete VP2 sequences to produce a total data set of 343 sequences, with the different host species shown in Figure 1. A maximum likelihood (ML) phylogenetic tree of these sequences was obtained using PhyML (version 3.0) [46], and rooted as previously shown in a (relaxed) molecular clock analysis [28]. For this analysis, we utilized the GTR+I+Γ_4 model of nucleotide substitution and a combination of subtree pruning and regrafting (SPR) and nearest neighbor interchange (NNI) branch swapping. The robustness of individual nodes on the phylogeny was estimated using bootstrap resampling, in this case, utilizing 1000 replicate ML trees and NNI branch swapping. Finally, to determine the occurrence of parallel mutations in VP2, all amino acid changes were mapped onto the ML phylogeny using the parsimony procedure available in the PAUP* package (version 4) [47].

Host adaptation studies

Viruses chosen for host adaptation studies included: (i) the pandemic CPV-2 prototype dog strain, CPV-2/Dog/NY/CPV-d/79 (CPV-d) (GenBank accession M23255), and (ii) FPV/Raccoon/TX/Rac3/78 (Rac3) (GenBank accession KM624023), an FPV-like virus isolated from a raccoon in 1978 that contains all the major capsid residues that are normally associated with FPV, except that it has VP2 residue 323-Asn, one of two mutations (i.e., 323-Asp to Asn and 93-Lys to Asn) that control the FPV host range for dog cells [9,29]. As a consequence, the Rac3 strain may represent a natural evolutionary 'intermediate' leading to CPV-2. We also analyzed (iii) CPV/Raccoon/VA/118-A/07 (Rac118) (GenBank accession JN867610), the prototype raccoon CPV strain first isolated in 2007 [29]. To determine if some of the mutations seen in Rac118 could be recapitulated in other raccoon CPV viruses, we also analyzed CPV/Raccoon/334-A/CA/10 (Rac334) (GenBank accession JX475261), a virus that has the signature VP2

300-Asp observed in Rac118 but differed at VP2 positions 224, 232, and 305 (Table 2), and in its phylogenetic background (Figure 1). Isolate Rac334 also differs from other CPV-like raccoon viruses in possessing 426-Asp rather than 426-Asn and is thus antigenically distinct (formerly described as 'CPV-2b-like') (Table 2). An amino acid alignment of the VP2 proteins of CPV-d, Rac3, Rac118, and Rac334 is shown in Figure S3.

The three raccoon viruses (Rac3, Rac118, and Rac334) were isolated by inoculation of clarified supernatant of original tissue homogenate into Nordon Laboratory feline kidney (NLFK) cell culture. Viruses were passaged one additional time to generate stock viruses and viral DNA was extracted using a QIAamp DNA mini kit (Qiagen, Valencia, CA). VP2 sequences were amplified using a GoTaq DNA polymerase kit (Promega, Madison, WI) and compared to the original tissue-derived VP2 sequences to determine whether any additional mutations had occurred. Stock viruses were stored at −80°C and titrated by $TCID_{50}$ in NLFK cells and the capsid titers were determined by hemagglutination assay as previously described [48]. CPV-2 (CPV-d) was derived from an infectious plasmid clone [49] by transfection of plasmid DNA into NLFK cell culture using Lipofectamine 2000 (Life Technologies, Carlsbad, CA) according to the manufacturer's instructions.

Viruses were passaged in six different cell lines which were derived from members of the order Carnivora, the normal hosts for these parvoviruses: domestic cat (*Felis catus*) kidney (NLFK), domestic dog (*Canis lupus familiaris*) tumor (A72), gray fox (*Urocyon cinereoargenteus*) lung (FoLu), American mink (*Neovision vison*) lung (Mv1Lu), raccoon (*Procyon lotor*) uterus (Pl1Ut), and domestic ferret (*Mustelo putorius furo*) brain (Mpf) cells. All cell lines (except NLFK cells) were obtained from the American Type Culture Collection (ATCC; Manassas, VA) and grown in minimum essential medium supplemented with 5–10% fetal bovine serum, 400 units/mL penicillin, 400 μg/mL streptomycin, and 1 μg/mL amphotericin B (Sigma, St. Louis, MO) in a 5% CO_2 atmosphere. Viruses were inoculated into each of the cell lines at the time of seeding ($\sim 1 \times 10^5$ cells/mL) using a multiplicity of infection (MOI) of 1 $TCID_{50}$ in a 10 cm^2 well (6-well plate) and incubated until the culture was confluent. The monolayer was then washed five times with sterile PBS to remove any residual virus, trypsinized, and the entire culture (1 mL) was transferred into a 75 cm^2 flask containing 19 mLs of media. Once the cell monolayer was confluent or showing substantial cytopathic effects prior to confluency, the supernatant (passage 2) was harvested and then used as inoculum for the next passage (passage 3). For passages 3–20, viruses were transferred at weekly intervals by inoculating 200 μL of supernatant into a freshly seeded 10 cm^2 well. Supernatants from passages 2, 5, 10 and 20 were examined for viral DNA and, if present, the VP2 gene was amplified by PCR and sequenced. VP2 sequences from passages 2, 5, 10, and 20 were then aligned to identify the temporal expression of mutations.

Single growth curve, competition, and relative infectivity assays

For single growth curve (multi-step) analysis, non-passaged (passage 0 or p0) and terminally passaged (passage 20 or p20 in Mpf ferret cells) CPV-2 were used. Titers of each stock was determined by $TCID_{50}$ in NLFK cells and used to calculate inoculums (MOI of 0.005) for experiments. All prepared inoculums were then back titrated in both NLFK and Mpf cells to confirm their accuracy. For the growth curves, Mpf cells were seeded at a density of 1×10^5 cells/mL in a 4.0 cm^2 format (12-well plate) and infected with $\sim$1000 $TCID_{50}$ of p0 CPV-2 or p20 Mpf CPV-2. For direct comparison between the growth curves of the p0 and p20 viruses, 12-well plates were seeded from the same flasks of Mpf cells to limit variability in cell density. Wells were harvested daily for 6 days and frozen at −80°C until processing. Titrations ($\log_{10} TCID_{50}$/mL) were then performed in NLFK cells in a 96-well plate format by an immunofluorescence assay. Briefly, on day 3 post-infection, cells were fixed with 10% formalin, washed three times with sterile PBS, and incubated with 50 μL of a 1:1500 dilution of a polyclonal rabbit anti-CPV VP1/VP2 antibody for 1 hr. The wash steps were repeated, and then the cells were incubated with 50 μL of a 1:4000 dilution of an Alexa Fluor 488 goat anti-rabbit IgG (H+L) antibody (Life Technologies) for 1 hr, followed by a final wash series. Immunofluorescence was analyzed using a Nikon Eclipse TE300 inverted fluorescence microscope equipped with a Hammamatsu OrcaER digital camera (Nikon Corporation, Tokyo, Japan).

For competition assays in Mpf cells, the same virus-cell combinations mentioned above (p0 stock CPV-2 and CPV-2 passaged 20 times in Mpf cells) were used. Cells were seeded at the same densities as above and then cells were infected with p0:p20 ratios of CPV-2 in the following formats: i) 1:1 ratio (1000 $TCID_{50}$: 1000 $TCID_{50}$), ii) 10:1 ratio (1800 $TCID_{50}$: 200 $TCID_{50}$), and iii) 1:10 ratio (200 $TCID_{50}$: 1800 $TCID_{50}$). Controls consisted of cells infected with each virus on their own to ensure no mutations occurred during passage. On days 2, 4, and 6, wells were harvested and DNA was extracted using a QIAamp DNA mini kit (Qiagen) according to the manufacturer's protocols. Key fixed mutations of difference between the p0 and p20 stocks (VP2 positions 375 and 562) were then analyzed at days 2, 4, and 6, and chromatograms of those residues were visualized using Geneious R7 software (Biomatters Ltd., Auckland, New Zealand).

For relative infectivity assays by immunofluorescence, domestic cat (NLFK), dog (A72), and ferret (Mpf) cells were used. Cells were seeded at a density of 1×10^5 cells/mL in a 4.0 cm^2 format (12-well plate) and infected with $\sim$1000 $TCID_{50}$ of either non-passaged Rac118 stock (containing VP2 300-Asp) or Rac118 passaged 20 times in A72 cells (which contains VP2 300-Gly). This virus-cell line combination was chosen for further analysis due to the observed blocks to infection with non-passaged Rac118 in dog cells, and that Rac118 also mutated at position 300 during passage in ferret cells, but not cat cells. Plates were set up in duplicate and fixed and stained on DPI 3 and DPI 6 (Figure 5E). Fixation, staining, and immunofluorescence were carried out as stated above except volumes were increased because of the 12-well plate format.

Transferrin receptor sequencing

The complete TfR sequences of the six host species used in adaptation experiments were obtained by RT-PCR of mRNA isolated from their cell lines (A72, FoLu, Mpf, Mv1Lu, NLFK, Pl1Ut) as previously described [28]. All cDNA sequences of the TfRs were amplified using a SuperScript III One-Step RT-PCR System with Platinum Taq (Invitrogen, Carlsbad, CA). For amplification of the entire TfR, primers to non-coding regions of the transcript were used to ensure the entire open reading frame was determined as previously described (primers available from authors upon request) [20]. The deduced amino acid sequences of the complete TfR of the six hosts were aligned using Clustal Omega (http://www.ebi.ac.uk/Tools/msa/clustalo/) and examined for potential regions or residues of conservation or diversity in or near the region where parvoviruses are known to bind (apical domain and adjacent areas) (Figures S1 and S2) [18,21]. Additional TfR cDNAs from other carnivores (species not used in the adaptation studies: gray wolf and Arctic fox) were amplified and sequenced using the same protocol. New TfR sequences

obtained from this study have been submitted to GenBank under the accession numbers KJ813896-KJ813902 (Table S2).

Molecular modeling of viruses and transferrin receptors

To better understand how the viral mutations observed during host adaptation, especially sequential mutations arising during passage in specific hosts, may be involved in determining infectivity, their spatial locations were visualized using the PyMOL Molecular Graphics System, Version 1.5.0.4 Schrödinger, LLC. For this analysis, we used the structures of the 300-Asp mutant of CPV-2 (PDB 1IJS). Amino acid changes in the capsid were made with the mutagenesis function in PyMOL. To analyze the predicted three-dimensional structures of the host TfRs, receptor models were made using the Protein Homology/AnalogY Recognition Engine 2 (Phyre2) web-based server available at http://www.sbg.bio.ic.ac.uk/~phyre2 [50]. The template sequence used to create the carnivore TfR models was the human TfR ectodomain crystal structure (PDB 1CX8) [17] and the dog models shown in Figure S2 were predicted using the Intensive Modeling option in Phyre2. For the dog TfR models, 639/770 residues (83%) were modeled at >90% accuracy, due to the incorporation of the cytoplasmic, transmembrane, and stalk domains (131 residues; 17%). TfR models were also visualized using PyMOL.

Supporting Information

Figure S1 Amino acid alignment of the complete TfRs of the six carnivore hosts used in the experimental evolution studies. The six domains of the TfR (cytoplasmic, transmembrane, stalk, protease, apical, and helical) [17] are indicated atop the alignment. Residues that are variable are highlighted, with each alternate residue shown in royal blue, cyan, yellow, or red. See Table S2 for GenBank accession numbers of each host TfR.
(TIF)

Figure S2 Amino acid alignment of the TfR apical domains of the six carnivore hosts used in the experimental evolution studies and the putative structural location of changes in the domestic dog model of the apical domain. (A) Amino acid alignment focusing on residues 197–393 (based on domestic dog numbering) which constitutes the apical domain, the region of the ectodomain involved in parvovirus binding [18]. Residues that are variable are highlighted, with each alternate residue shown in royal blue, cyan, yellow, or red. Positions that have either three or four different residues among the six species are numbered. The entire column for the 222-Leu residue, shown to be critical in parvovirus binding [21], is highlighted in cyan. The glycosylation site (NLT) at residue 384 in the domestic dog, coyote, and gray wolf TfRs that blocks FPV binding is highlighted in bright green. (B) Structural mapping of the sequence changes in the TfRs of the six hosts analyzed. The ribbon model of the domestic dog apical domain is shown, with residues of divergence among the six hosts color-coded as in panel A, with sites with two, three, or four mutations shown in royal blue, yellow, and red, respectively. (C) Surface rendition of a monomer of the domestic dog TfR homodimer, with the apical domain highlighted in light grey. (D) Side view (~45° counter-clockwise rotation from C) of the apical domain highlighting areas of divergence and/or importance.
(TIF)

Figure S3 Amino acid alignment of the VP2 protein of the parvoviruses used in the experimental evolution studies (CPV-d, Rac3, Rac118, Rac334). Non-passaged (original) sequences are shown, with residues of divergence highlighted.
(TIF)

Table S1 New full-length VP2 sequences of parvoviruses obtained from wild carnivores during this study. For each virus, the identification (ID) number, host species, county, state, and date of collection is shown, along with a GenBank accession number.
(TIF)

Table S2 New carnivore TfR sequences obtained during this study. For each carnivore species, its taxonomic classification and the source of mRNA is shown, along with a GenBank accession number.
(TIF)

Acknowledgments

We thank Justin Brown, Tom DeLiberto, Dennis Voronin, and Tom Zhao for technical and/or logistical support and United States Department of Agriculture-Animal and Plant Health Inspection Service-Wildlife Services and North Dakota Game and Fish Department personnel for collecting wild carnivore samples. We also thank Kimberlee Beckman, Alaska Department of Fish and Game, Juneau, Alaska, for providing gray wolf and Arctic fox tissues for TfR cDNA cloning.

Author Contributions

Conceived and designed the experiments: ABA ECH CRP. Performed the experiments: ABA ECH. Analyzed the data: ABA ECH CRP. Contributed reagents/materials/analysis tools: DJK AO EAH DMG. Wrote the paper: ABA ECH CRP.

References

1. Parrish CR, Holmes EC, Morens DM, Park EC, Burke DS, et al. (2008) Cross-species virus transmission and the emergence of new epidemic diseases. Microbiol Mol Biol Rev 72: 457–470.
2. Parrish CR, Kawaoka Y (2005) The origins of new pandemic viruses: the acquisition of new host ranges by canine parvovirus and influenza A viruses. Annu Rev Microbiol 59: 553–586.
3. Kuiken T, Holmes EC, McCauley J, Rimmelzwaan GF, Williams CS, et al. (2006) Host species barriers to influenza virus infections. Science 312: 394–397.
4. Parrish CR (1999) Host range relationships and the evolution of canine parvovirus. Vet Microbiol 69: 29–40.
5. Parrish CR, O'Connell PH, Evermann JF, Carmichael LE (1985) Natural variation of canine parvovirus. Science 230: 1046–1048.
6. Parrish CR, Have P, Foreyt WJ, Evermann JF, Senda M, et al. (1988) The global spread and replacement of canine parvovirus strains. J Gen Virol 69: 1111–1116.
7. Berns KI, Parrish CR (2013) Chapter 57: Parvoviridae. In: Knipe DM, Howley PM, editors. Field's Virology, sixth edition, Lippincott Williams and Wilkins, Philadelphia, Pennsylvania. pp. 1768–1791.
8. Tsao J, Chapman MS, Agbandje M, Keller W, Smith K, et al. (1991) The three-dimensional structure of canine parvovirus and its functional implications. Science 251: 1456–1464.
9. Chang SF, Sgro JY, Parrish CR (1992) Multiple amino acids in the capsid structure of canine parvovirus coordinately determine the canine host range and specific antigenic and hemagglutination properties. J Virol 66: 6858–6867.
10. Strassheim ML, Gruenberg A, Veijalainen P, Sgro JY, Parrish CR (1994) Two dominant neutralizing antigenic determinants of canine parvovirus are found on the threefold spike of the virus capsid. Virology 198: 175–184.
11. Parker JS, Parrish CR (1997) Canine parvovirus host range is determined by the specific conformation of an additional region of the capsid. J Virol 71: 9214–9222.

12. Govindasamy L, Hueffer K, Parrish CR, Agbandje-McKenna M (2003) Structures of host range-controlling regions of the capsids of canine and feline parvoviruses and mutants. J Virol 77: 12211–12221.
13. Hueffer K, Govindasamy L, Agbande-McKenna M, Parrish CR (2003) Combinations of two capsid regions controlling canine host range determine canine transferrin receptor binding by canine and feline parvoviruses. J Virol 77: 10099–10105.
14. Parker JS, Murphy WJ, Wang D, O'Brien SJ, Parrish CR (2001) Canine and feline parvoviruses can use human or feline transferrin receptors to bind, enter, and infect cells. J Virol 75: 3896–3902.
15. Hueffer K, Parker JS, Weichert WS, Geisel RE, Sgro JY, et al. (2003) The natural host range shift and subsequent evolution of canine parvovirus resulted from virus-specific binding to the canine transferrin receptor. J Virol 77: 1718–1726.
16. Luck AN, Mason AB (2012) Transferrin-mediated cellular iron delivery. Curr Top Membr 69: 3–35.
17. Lawrence CM, Ray S, Babyonyshev M, Galluser R, Borhani DW, et al. (1999) Crystal structure of the ectodomain of human transferrin receptor. Science 286: 779–782.
18. Palermo LM, Hueffer K, Parrish CR (2003) Residues in the apical domain of the feline and canine transferrin receptors control host-specific binding and cell infection of canine and feline parvoviruses. J Virol 77: 8915–8923.
19. Palermo LM, Hafenstein SL, Parrish CR (2006) Purified feline and canine transferrin receptors reveal complex interactions with the capsids of canine and feline parvoviruses that correspond to their host ranges. J Virol 80: 8482–8492.
20. Kaelber JT, Demogines A, Harbison CE, Allison AB, Goodman LB, et al. (2012) Evolutionary reconstructions of the transferrin receptor of caniforms supports canine parvovirus being a re-emerged and not a novel pathogen in dogs. PLoS Pathog 8(5): e1002666.
21. Goodman LB, Lyi SM, Johnson NC, Cifuente JO, Hafenstein SL, et al. (2010) Binding site on the transferrin receptor for the parvovirus capsid and effects of altered affinity on cell uptake and infection. J Virol 84: 4969–4978.
22. Barker IK, Parrish CR (2001) Parvovirus infections. In: Williams ES, Barker IK, editors. Infectious Diseases of Wild Mammals, third edition, Blackwell Publishing Co., Ames, IA. pp. 131–146.
23. Steinel A, Munson L, van Vuuren M, Truyen U (2000) Genetic characterization of feline parvovirus sequences from various carnivores. J Gen Virol 81: 345–350.
24. Steinel A, Parrish CR, Bloom ME, Truyen U (2001) Parvovirus infections in wild carnivores. J Wildl Dis 37: 594–607.
25. Neuvonen E, Veijalainen P, Kangas J (1982) Canine parvovirus infection in housed raccoon dogs and foxes in Finland. Vet Rec 110: 448–449.
26. Veijalainen P (1988) Characterization of biological and antigenic properties of raccoon dog and blue fox parvoviruses: a monoclonal antibody study. Vet Microbiol 16: 219–230.
27. Veijalainen PM, Neuvonen E, Niskanen A, Juokslahti T (1986) Latex agglutination test for detecting feline panleukopenia virus, canine parvovirus, and parvoviruses of fur animals. J Clin Microbiol 23: 556–559
28. Allison AB, Kohler DJ, Fox KA, Brown JD, Gerhold RW, et al. (2013) Frequent cross-species transmission of parvoviruses among diverse carnivore hosts. J Virol 87: 2342–2347.
29. Allison AB, Harbison CE, Pagan I, Stucker KM, Kaelber JT, et al. (2012) The role of intermediate hosts in the cross-species transmission and emergence of a pandemic virus. J Virol 86: 865–872.
30. Barun A, Hanson CC, Campbell KJ, Simberloff D (2011) A review of small Indian mongoose management and eradications on islands. In: Veitch CR, Clout MN, Towns DR, editors. Island invasives: eradication and management. International Union for Conservation of Nature, Gland, Switzerland. pp. 17–25.
31. Hong C, Decaro N, Desario C, Tanner P, Pardo MC, et al. (2007) Occurrence of canine parvovirus type 2c in the United States. J Vet Diagn Invest 19: 535–539.
32. Simpson AA, Chandrasekar V, Hébert B, Sullivan GM, Rossmann MG, et al. (2000) Host range and variability of calcium binding by surface loops in the capsids of canine and feline parvoviruses. J Mol Biol 300: 597–610.
33. Hoelzer K, Shackelton LA, Parrish CR, Holmes EC (2008) Phylogenetic analysis reveals the emergence, evolution and dispersal of carnivore parvoviruses. J Gen Virol 89: 2280–2289.
34. Ikeda Y, Nakamura K, Miyazawa T, Takahashi E, Mochizuki M (2002) Feline host range of canine parvovirus: recent emergence of new antigenic types in cats. Emerg Infect Dis 8: 341–346.
35. Llamas-Saiz AL, Agbandje-McKenna M, Parker JS, Wahid AT, Parrish CR (1996) Structural analysis of a mutation in canine parvovirus which controls antigenicity and host range. Virology 225: 65–71.
36. Hafenstein S, Palermo LM, Kostyuchenko VA, Xiao C, Morais MC, et al. (2007) Asymmetric binding of transferrin receptor to parvovirus capsids. Proc Natl Acad Sci U S A 104: 6585–6589.
37. Barbis DP, Chang SF, Parrish CR (1992) Mutations adjacent to the dimple of the canine parvovirus capsid structure affect sialic acid binding. Virology 191: 301–308.
38. Truyen U, Evermann JF, Vieler E, Parrish CR (1996) Evolution of canine parvovirus involved loss and gain of feline host range. Virology 215: 186–189.
39. Nowak RM (2005) Walker's Carnivores of the World. Baltimore, Maryland: Johns Hopkins University Press. 313 p.
40. Goss LJ (1948) Species susceptibility to the viruses of Carre and feline enteritis. Am J Vet Res 9: 65–68.
41. Phillips CE (1943) Haemorrhagic enteritis in the arctic blue fox caused by the virus of feline enteritis. Can J Comp Med 7: 33–35.
42. Hoelzer K, Parrish CR (2010) The emergence of parvoviruses of carnivores. Vet Res 41: 39.
43. Woolhouse ME, Taylor LH, Haydon DT (2001) Population biology of multihost pathogens. Science 292: 1109–1112.
44. Qin Q, Loeffler IK, Li M, Tian K, Wei F (2007) Sequence analysis of a canine parvovirus isolated from a red panda (*Ailurus fulgens*) in China. Virus Genes 34: 299–302.
45. Truyen U, Müller T, Heidrich R, Tackmann K, Carmichael LE (1998) Survey on viral pathogens in wild red foxes (*Vulpes vulpes*) in Germany with emphasis on parvoviruses and analysis of a DNA sequence from a red fox parvovirus. Epidemiol Infect 121: 433–440.
46. Guindon S, Dufayard J-F, Lefort V, Anisimova M, Hordijk W, et al. (2010) New algorithms and methods to estimate maximum-likelihood phylogenies: assessing the performance of PhyML 3.0. Syst Biol 59: 307–321.
47. Swofford DL (2003) PAUP*: Phylogenetic analysis using parsimony (*and other methods) version 4.0. Sinauer Associates, Inc., Sunderland, Massachusetts.
48. Truyen U, Parrish CR (1992) Canine and feline host ranges of canine parvovirus and feline panleukopenia virus: distinct host cell tropisms of each virus *in vitro* and *in vivo*. J Virol 66: 5399–5408.
49. Parrish CR (1991) Mapping specific functions in the capsid structure of canine parvovirus and feline panleukopenia virus using infectious clones. Virology 183: 195–205.
50. Kelley LA, Sternberg MJ (2009) Protein structure prediction on the Web: a case study using the Phyre server. Nat Protoc 4: 363–371.
